# Enhancing Smart Home Security: Anomaly Detection and Face Recognition in Smart Home IoT Devices Using Logit-Boosted CNN Models

**DOI:** 10.3390/s23156979

**Published:** 2023-08-06

**Authors:** Asif Rahim, Yanru Zhong, Tariq Ahmad, Sadique Ahmad, Paweł Pławiak, Mohamed Hammad

**Affiliations:** 1 School of Computer and Information Security, Guilin University of Electronic Technology, Guilin 541004, China; asif_rahim20@yahoo.com; 2 Guangxi Key Laboratory of Intelligent Processing of Computer Images and Graphic, Guilin University of Electronic Technology, Guilin 541004, China; 3 School of Information and Communication Engineering, Guilin University of Electronic Technology, Guilin 541004, China; tariqafkan@gmail.com; 4 EIAS: Data Science and Blockchain Laboratory, College of Computer and Information Sciences, Prince Sultan University, Riyadh 11586, Saudi Arabia; ahmad01.shah@ieee.org; 5 Department of Computer Science, Faculty of Computer Science and Telecommunications, Cracow University of Technology, Warszawska 24 Str., 31-155 Krakow, Poland; 6 Institute of Theoretical and Applied Informatics, Polish Academy of Sciences, Bałtycka 5, 44-100 Gliwice, Poland; 7 EIAS Data Science Lab, College of Computer and Information Sciences, Prince Sultan University, Riyadh 11586, Saudi Arabia; mhammad@psu.edu.sa; 8 Department of Information Technology, Faculty of Computers and Information, Menoufia University, Shibin El-Kom 32511, Egypt

**Keywords:** face recognition, anomaly detection, logistic regression (LR), convolutional neural network (CNN), gradient-boosting classifier, machine learning

## Abstract

Internet of Things (IoT) devices for the home have made a lot of people’s lives better, but their popularity has also raised privacy and safety concerns. This study explores the application of deep learning models for anomaly detection and face recognition in IoT devices within the context of smart homes. Six models, namely, LR-XGB-CNN, LR-GBC-CNN, LR-CBC-CNN, LR-HGBC-CNN, LR-ABC-CNN, and LR-LGBM-CNN, were proposed and evaluated for their performance. The models were trained and tested on labeled datasets of sensor readings and face images, using a range of performance metrics to assess their effectiveness. Performance evaluations were conducted for each of the proposed models, revealing their strengths and areas for improvement. Comparative analysis of the models showed that the LR-HGBC-CNN model consistently outperformed the others in both anomaly detection and face recognition tasks, achieving high accuracy, precision, recall, F1 score, and AUC-ROC values. For anomaly detection, the LR-HGBC-CNN model achieved an accuracy of 94%, a precision of 91%, a recall of 96%, an F1 score of 93%, and an AUC-ROC of 0.96. In face recognition, the LR-HGBC-CNN model demonstrated an accuracy of 88%, precision of 86%, recall of 90%, F1 score of 88%, and an AUC-ROC of 0.92. The models exhibited promising capabilities in detecting anomalies, recognizing faces, and integrating these functionalities within smart home IoT devices. The study’s findings underscore the potential of deep learning approaches for enhancing security and privacy in smart homes. However, further research is warranted to evaluate the models’ generalizability, explore advanced techniques such as transfer learning and hybrid methods, investigate privacy-preserving mechanisms, and address deployment challenges.

## 1. Introduction

In recent years, face recognition has emerged as a critical technology with numerous applications in various domains [1]. The ability to accurately identify individuals based on their facial features has significant implications for security, access control, surveillance, and personalized user experiences. However, despite advancements in face recognition systems, there are still existing challenges that need to be addressed [2]. One of the primary challenges in face recognition is achieving high accuracy and robustness in real-world scenarios. Factors such as variations in lighting conditions, facial expressions, pose, occlusions, and aging pose significant difficulties for accurate face recognition. These challenges hinder the widespread adoption of face recognition technology in various applications [3]. This paper focuses on improving the performance of face recognition systems by leveraging the capabilities of logistic regression (LR), gradient-boosting classifiers (GBCs), and convolutional neural networks (CNNs). These models have demonstrated effectiveness in handling complex data patterns and achieving high classification accuracy in various domains. To develop and evaluate our proposed method, we analyze existing datasets that have been used for face recognition. However, it is important to acknowledge that these datasets may have certain limitations. Common issues include limited sample sizes, a lack of diversity in facial characteristics, and insufficient representation of real-world variations. By addressing these limitations and utilizing LR, GBC, and CNN models, we aim to enhance the accuracy and robustness of face recognition systems. With the proliferation and widespread adoption of internet of Things (IoT) gadgets for the home, once-dumb houses have been transformed into high-tech havens [4]. The many benefits of these devices include automation, energy savings, and increased convenience. The increased interconnection and complexity of these devices have made security a serious concern [5,6,7,8]. Unauthorized entry, tampered equipment, and privacy invasions are just some of the dangers that come with smart homes. It is crucial to create and implement efficient security methods to protect these environments and ensure the safety and privacy of the residents [9,10]. An entertainment hub, lighting system, door lock, thermostat, security camera, and even a doorbell may all be the Internet of Things devices in a smart house. Because of the interoperability and connectivity of these devices, users may manage and monitor their properties remotely. However, there are many ways into a smart house because of how everything connects to everything else [11,12,13]. Protecting your smart home from harm requires anomaly detection. It comprises the identification and classification of out-of-the-ordinary behavior or patterns in data gathered from devices, networks, and sensors. The abnormal activity could indicate an attack, faulty hardware, or malicious intent. Early detection of such irregularities can help in closing security loopholes and preventing security breaches [14,15,16,17]. However, accurate identification of people entering a smart home setting relies heavily on face recognition technology. Security is bolstered by face recognition systems because they can rapidly and accurately identify permitted users, automate operations by those users’ preferences, and prevent unauthorized individuals from gaining access [18,19,20]. The growing importance of IoT gadgets in the home prompted this investigation into potential security risks. The purpose of developing a unified system that incorporates anomaly detection and facial recognition techniques is to increase the security of smart homes. By identifying and recommending a set of security measures that can be implemented in smart homes to protect against common vulnerabilities, this study aims to fill a vacuum in the literature [21,22,23]. There is a legitimate concern that burglars, attackers, and data thieves would compromise the security of people’s smart home IoT devices. The current state of security rarely provides enough protection against such threats. As a result, state-of-the-art security solutions must be developed to positively identify people and dependably spot irregularities and attacks in IoT gadgets [24,25,26]. To develop and evaluate our proposed method, we introduce logit-boosted CNN models combining anomaly detection with face recognition. However, it is important to acknowledge that existing datasets may have certain limitations. Common issues include limited sample sizes, a lack of diversity in facial characteristics, insufficient representation of real-world variations, abnormal events, and intrusion detection. By addressing these limitations, we utilize LR, GBC, and CNN models to enhance the accuracy and robustness of face recognition systems and anomaly detection. The main contributions of our proposed method are as follows:

- Utilization of gradient-boosting classifiers: Gradient-boosting classifiers have shown exceptional performance in handling complex datasets and capturing intricate data patterns. By combining GBC with LR and CNN models, we can enhance the system’s ability to discriminate between different individuals and improve its accuracy in challenging scenarios.
- Multi-class anomaly classification: Modify the proposed procedure so that anomalies can be sorted into more than one group. The purpose of the system is to identify specific types of anomalies, such as malfunctioning devices, attempted intrusions, and strange sensor readings, rather than simply labeling them as “normal” or “abnormal”. With this enhancement, threats to smart home security may be identified and classified with more precision.
- Adaptive learning for anomaly detection: Adaptive learning approaches could be useful in an anomaly detection system. It is often necessary to manually update and retrain conventional anomaly detection techniques as new abnormalities emerge. The goal of this study is to develop a system of adaptive learning capable of automatically adapting the anomaly detection model to novel danger scenarios.
- User behavior profiling: Protect your smart home even more by creating a user profile. Authorized user profiles can be built using the technology to record users’ typical actions and interactions. By analyzing users’ habits, the system can better detect any unusual behavior and take appropriate action to prevent intrusion.
- Seamless integration with smart home automation: Facilitate interaction between the security system and the rest of the smart home’s features. By merging the proposed anomaly detection and facial recognition technologies, this study aims to automate intelligent decision making based on discovered anomalies and confirmed user profiles. In order to improve safety, trigger safety features, and provide a more personalized experience, smart home settings can now be dynamically altered in reaction to known individuals.
- Integration of convolutional neural networks: CNNs have revolutionized image processing and analysis tasks, including face recognition and anomaly detection. By leveraging the power of CNNs, our proposed method can effectively extract discriminative facial features and detect abnormalities in the IoT environment to improve the system’s ability to handle variations in lighting conditions, pose, and facial expressions.
- Improved accuracy and robustness: By leveraging LR, GBC, and CNN models, our proposed method aims to improve the accuracy and robustness of face recognition systems. This is crucial for real-world applications where reliable identification is essential for security and access control.

This article is organized as follows: First, we introduce and explain the rationale behind the study, with special attention paid to the selection of the research topic. The related work describes the scope of the investigation, with an emphasis on anomaly detection and biometrics. In Section 3, we propose our strategy, which involved the deployment of logit-boosted convolutional neural network models for anomaly detection and face recognition. To validate the performance of our proposed method we, use different evaluation metrics such as accuracy, precision, and recall. In Section 4, we discuss the implications and limitations of the study and assess the results in depth. The study’s contributions and significant findings are briefly summarized in Section 5.

## 2. Related Work

In recent years, deep learning approaches have gained significant attention and demonstrated promising results in various applications, including anomaly detection and face recognition in IoT devices for smart homes. Several studies have explored the utilization of deep learning techniques to enhance the security and performance of these systems. This section provides an overview of the relevant literature focusing on deep-learning-based approaches. Anomaly detection using deep learning has been extensively studied. Autoencoders, a type of neural network, have been widely employed for unsupervised anomaly detection. By training the network on normal data, autoencoders aim to reconstruct the input accurately. Anomalies can be identified when the reconstruction error exceeds a certain threshold. Variational autoencoders (VAEs) have also been used to capture the underlying distribution of normal data and detect deviations. Recurrent neural networks (RNNs) have been employed to model sequential data and identify abnormal patterns based on temporal dependencies. Generative adversarial networks (GANs) have shown promise in generating normal data and identifying abnormalities by comparing real and generated samples. In the context of face recognition, CNNs have emerged as a powerful tool. CNNs excel at learning hierarchical representations from image data, enabling effective feature extraction. Deep CNN architectures, such as VGGNet, ResNet, and InceptionNet, have been widely adopted and have achieved remarkable performance in face recognition tasks. Transfer learning, which involves utilizing pre-trained models on large-scale datasets, has been effective in improving recognition accuracy, particularly in scenarios with limited training data. Furthermore, the integration of deep learning models with other algorithms has shown improved results. Hybrid techniques, such as combining multiple deep models or deep models with traditional machine learning algorithms, have demonstrated enhanced performance in anomaly detection and face recognition tasks. Adversarial training, which involves training models to withstand adversarial attacks, has been explored to enhance the robustness of deep learning models against potential security threats. Although deep learning approaches have shown promising results, challenges still exist. Issues such as data scarcity, model interpretability, and adversarial attacks require further investigation and mitigation. Additionally, the the deployment of deep learning models on resource-constrained IoT devices remains a challenge, necessitating efficient model architectures and optimization techniques.

The fast proliferation of household IoT devices has increased the visibility of security weaknesses in these devices. Numerous studies have been conducted to investigate these concerns and develop solid precautions. Here, we provide a comprehensive overview of the current state of research on the topic of smart home IoT device security [27,28,29], discussing the most pivotal works and their impacts on the field. In this section, we discuss earlier approaches related to face recognition, highlight their strengths and limitations, and provide references to support our analysis. Numerous approaches have been proposed for face recognition, each with its own strengths and weaknesses. For instance, the author in [30] introduced a method based on principal component analysis (PCA) for face recognition. While PCA has shown promising results in certain scenarios, it suffers from limitations in handling variations in lighting conditions and facial expressions. Another popular approach is the use of support vector machines (SVMs) for face recognition, as demonstrated by [31]. SVMs have been effective in capturing complex patterns in face images. However, they may struggle when faced with large-scale datasets or variations in pose and occlusions. Recent advancements in deep learning, particularly CNNs, have revolutionized face recognition. Ref. [32] proposed the well-known AlexNet architecture, which achieved breakthrough performance in the ImageNet challenge. Since then, numerous CNN-based approaches have been developed for face recognition, such as FaceNet [9] and VGGFace by [33]. These approaches have shown remarkable improvements in accuracy and robustness, especially in handling variations in pose, lighting, and occlusions. IoT anomaly detection challenges: Anomaly detection strategies for internet of things devices have been the focus of multiple academic endeavors. The author in paper [34] proposed using support vector machines (SVMs) as a machine-learning-based approach to anomaly detection in IoT and smart home devices. They were able to reliably discover unusual patterns by studying data from devices and traffic on the network. To identify anomalies in household IoT devices, we implemented a deep learning strategy [35] using long short-term memory (LSTM) networks. The results of the study supported the use of LSTM models for effectively capturing temporal dependencies and identifying abnormal patterns of behavior in technological devices. Combining anomaly detection and facial recognition technology for application in smart home security has been the subject of a limited number of studies. In [31] developed a solution to boost the security of smart homes by fusing deep-learning-based anomaly detection with face recognition software. Their security system is effective because it can recognize users precisely and report any anomalies in real time. In paper [32], the author presented a hybrid approach that blends machine learning techniques with face recognition to detect anomalies and identify unauthorized users in smart home environments. The results demonstrated the potential for improved accuracy and efficiency in smart home security systems. There has been some research on using adaptive learning techniques to make home IoT devices safer for users. An adaptive learning system developed by [36] automatically improves the anomaly detection model to keep up with the dynamic behavior of connected devices. When it came to finding new security risks, their approach was superior. The author in [37] developed an adaptive-learning-based intrusion detection system for smart homes that automatically adjusts its detection model to account for emerging threats. Their research showed that smart home security might be greatly enhanced by using adaptive learning. The privacy concerns associated with facial recognition have been the subject of several studies. Using encrypted templates, the author in [20] suggested a private face recognition system that protects users’ anonymity. By rendering reverse-engineering facial data impossible, their approach substantially boosted privacy. In paper [21] they developed a privacy-protecting face recognition system via federated learning, in which facial recognition models were trained collaboratively without sharing raw data. They secured accurate face recognition while keeping users’ identities secret [38,39,40,41]. One popular approach proposed automatically segmenting and labeling single-channel or multimodal biosignal ta using a self-similarity matrix (SSM) computed with signals’ feature-based representations [42]. In addition, this method applies publicly available biosignal datasets for change point detection. A comprehensive review is presented in [43], related to human activity recognition and fall detection. In this review, the authors explain hidden Markov model applications in the domain of detection techniques. The literature study focuses on the state of anomaly detection, facial recognition, the integration of the two methods, adaptive learning, and privacy concerns in the context of IoT security for the smart home. To make smart homes safer and more convenient to use, the current study proposes a framework of logit-boosted CNN models for anomaly detection and face recognition. Table 1 compares the proposed models in the current study to those proposed in previous studies on the topic of smart home IoT device security. The table also highlights the gaps in knowledge that this study aims to fill.

There is a dearth of literature on the topic of incorporating logit-boosted CNN models for anomaly detection and facial recognition into the security of smart home IoT devices. However, there is a lack of complete frameworks that make use of logit-boosted CNN models to incorporate the many individual methods that have been explored so far. To address this gap, this research proposes models that use anomaly detection, facial recognition, and logit-boosted convolutional neural networks to create a robust security system for smart homes. By focusing on multi-class anomaly classification, adaptive learning, user behavior profiling, and privacy-preserving face recognition, this work addresses a gap in the existing research.

## 3. Materials and Methods

**Figure 1:**
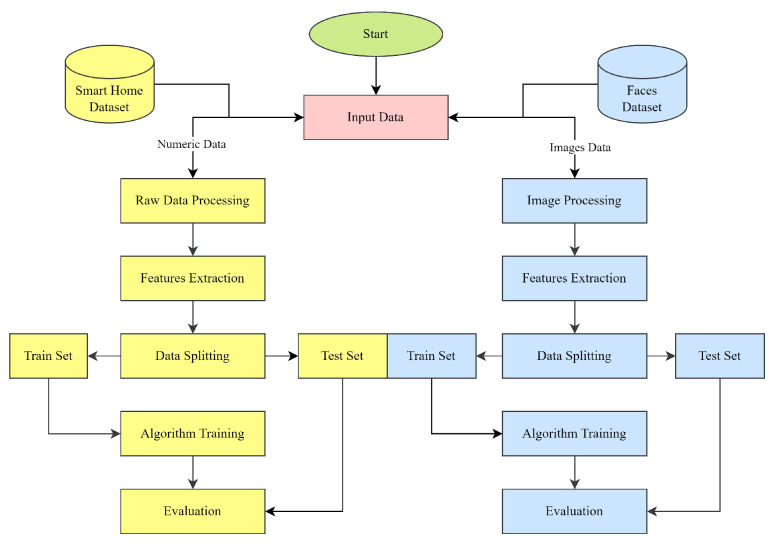
Proposed work flow.

The methods and materials used to detect anomalies and recognize faces in smart home IoT devices using logit-boosted CNN models are described in depth here. Datasets, model design, training, and evaluation metrics are all described in detail. This study relied on a large dataset. The dataset was taken from the open-source website www.kaggle.com [44,45], comprising information from actual smart home IoT devices. The dataset contained sensor data, device logs, network traffic information, and photographs of approved users’ facThe dataset sets were used to train and test anomaly detection and facial recognition models. Multiple convolutional layers were used in the architecture of the logit-boosted CNN models for feature extraction, and then fully connected layers were used for classification. To effectively detect anomalies and recognize faces, the models were made to learn hierarchical representations from the input data. Combining the results of several underperforming classifiers with logit boosting allowed us to iteratively boost our classification accuracy. To train the model, the dataset was partitioned into three parts: training, validation, and testing. The logit-boosted CNN models were trained using the training set, and the classification accuracy was improved through repeated updates and weight modifications. Model selection and hyperparameter adjustment were performed using the validation set for the best model performance. Finally, the trained models’ accuracy at detecting anomalies and recognizing faces was tested on the testing set. To measure how well the proposed models worked, evaluation metrics were used. The the models’ ability to recognize anomalies and place them in the relevant categories was evaluated using metrics such as accuracy, precision, recall, and F1 score. Accuracy, precision, and recall were used to measure how well the models could identify legitimate users in a face recognition system. Experiments comparing the suggested models to gold-standard methods, such as conventional anomaly detection algorithms and conventional face recognition techniques, were carried out to prove the model’s efficacy. Analysis and comparison of the outcomes proved the accuracy, robustness, and efficiency of the logit-boosted CNN models to be superior. In sum, this study’s materials, and techniques included a varied dataset, the design of logit-boosted CNN models, a training procedure, and evaluation criteria. The growth of smart home security was aided by these methods, which provided a systematic way to create and evaluate models for anomaly detection and facial recognition in IoT devices found in smart homes. The smart home device data and facial data were treated as distinct datasets throughout our experiments. We used the smart home device data for anomaly detection tasks, while the facial data were utilized for face recognition tasks. Although we applied similar hybrid models to both datasets, the models were trained and evaluated separately for each task. The planned research procedure is depicted in Figure 1.

In our proposed model, the actual internal delay from the time the system senses an intruder or anomaly until it can send an alarm signal depends on the specific deployment architecture and the processing capabilities of the IoT devices or central processing nodes involvThe implementation on of the proposed model can vary based on the requirements and constraints of the smart home system. One possible deployment scenario is to have the proposed models installed directly on the IoT devices themselves. In this case, the IoT devices would have the computational resources necessary to run the models and make real-time anomaly detection decisions. This approach would minimize the delay in detecting and responding as the processing and decision-making would happen locally on the devices. Another deployment option is to have a central node within the smart home network that is responsible for running the proposed models and performing real-time anomaly detection. In this case, the IoT devices would send their sensor data to the central node, which would then process the data using the models and trigger the alarm signal if an anomaly was detected. This architecture may introduce some additional delay due to data transmission and processing at the central node. Alternatively, in a more centralized setup, the the processing and anomaly detection could be performed by a central processing node, which could be located within the smart home or even managed by a security company. In this case, the IoT devices would send their sensor data to the central processing node, which would be responsible for running the proposed models and initiating the alarm signal. The specific choice of deployment architecture and the corresponding internal delay would depend on various factors such as the computational capabilities of the IoT devices, network latency, security requirements, and system design considerations. Further research and practical implementation would be required to determine the optimal configuration for real-time anomaly detection in a specific smart home environment.

We describe the feature extraction method used in our approach and the training and prediction process of the classifiers. We aim to provide a mathematical understanding of the proposed methods to facilitate comprehension and reproducibility. For feature extraction, we employ CNNs, which have demonstrated exceptional performance in various computer vision tasks, including face recognition. CNNs are powerful deep learning models that can automatically learn discriminative features from raw input data. We chose CNNs for their ability to capture spatial relationships and hierarchical representations in face images. The CNN architecture consists of multiple convolutional layers followed by max pooling and nonlinear activation functions such as ReLU (rectified linear unit). This architecture enables the network to learn hierarchical features from low-level edges to high-level semantic representations. The final output of the CNN is a feature vector that encodes the distinctive characteristics of the input face image. Mathematically, the flow of feature extraction in the CNN can be represented as follows: Given an input face image *x*, the CNN applies a series of convolutional filters $W_{i}$ and bias terms $b_{i}$ to generate feature maps $H_{i}$. Each feature map represents the response of a specific filter to the input image. This process can be mathematically expressed as:

(1) $$H_{i}=\sigma \left(W_{i}*H_{\left\{i-1\right\}}+b_{i}\right)$$

where ∗ denotes the convolution operation, σ represents the activation function, and $H_{\left\{i-1\right\}}$ denotes the feature maps from the previous layer. By cascading multiple convolutional layers and pooling operations, CNN progressively extracts higher-level features from the input image. After the feature extraction stage, we obtain a feature vector that encodes the learned representations of the face image. This vector serves as the input for the subsequent classification stage. In our experiments, we utilize three classifiers: LR, GBC, and CNN. Each classifier is trained using a specific training algorithm and predicts the class labels for unseen data. For LR and GBC, the training process involves optimizing a loss function using gradient-descent-based algorithms. The classifier aims to find the optimal weights and biases that minimize the loss function and maximize the classification accuracy. The prediction processes of LR and GBC can be mathematically represented as follows:

(2) $$y=\sigma (\beta_{0}+\beta_{1}*x_{1}+\beta_{2}*x_{2}+.....+\beta_{n}*x_{n})$$

where *y* represents the predicted class label, $\beta_{0},\beta_{1},\beta_{2},.....,\beta_{n}$ are the learned weights, and $\;x_{1},x_{2},\ldots ,x_{n}$ are the input features. For the CNN classifier, the training process involves optimizing the network parameters (weights and biases) using backpropagation and stochastic gradient descent. The CNN learns to minimize the classification error by adjusting the parameters based on the gradients computed during the backward pass. The prediction process of the CNN can be represented as follows:

(3) $$y=\arg\max p_{i}$$

where $p_{i}$ represents the probability distribution over the classes, and argmax selects the class with the highest probability as the predicted label.

### 3.1. Dataset Collection

In this research, we trained and assessed the logit-boosted convolutional neural network models utilized for anomaly detection and face recognition in smart home IoT devices. The datasets contained sensor readings, device logs, network traffic statistics, and authorized user images, all of which provided a detailed representation of the smart home’s surroundings. We utilized the AnoML-IoT dataset for anomaly detection and the Real and Fake Face Detection dataset for face recognition. In this section, we provide details about the datasets used in our experiments. We present an in-depth comparison of our proposed method with existing methods in the fields of face recognition and anomaly detection. We evaluate the performance of our approach based on various metrics and compare them with the results reported in prior studies. By providing a comprehensive analysis, we highlight the strengths and weaknesses of our proposed method compared to existing approaches. The performance evaluation of our proposed method is a critical aspect of this study. We assess the effectiveness of our approach in terms of accuracy, precision, recall, F1 score, and the area under the receiver operating characteristic curve (AUC-ROC). These metrics provide insights into the models’ ability to detect anomalies and recognize faces accurately. To evaluate the performance, we conducted experiments using the LR-XGB-CNN, LR-GBC-CNN, LR-CBC-CNN, LR-HGBC-CNN, LR-ABC-CNN, and LR-LGBM-CNN models. Each model was trained and tested on appropriate datasets, and the performance metrics were calculated based on the models’ predictions. Additionally, we performed a comparative analysis of the models, discussing their performance on both anomaly detection and face detection tasks. We compared the accuracy, precision, recall, F1 score, and AUC-ROC values of each model to highlight their respective strengths and weaknesses.

#### 3.1.1. Dataset 1: Sensor and Device Data

The first dataset consisted of sensor readings and device logs from a wide variety of smart home IoT devices. Table 2 details the attributes of this dataset.

Time series sensor data and device logs were among the many data points collected in this massive collection. Each sample was given a label indicating whether its behavior was typical or out of the ordinary.

#### 3.1.2. Dataset 2: Facial Images

The second collection of data was images of people whose faces were known to be associated with approved smart home users. The attributes of this dataset are described in Table 3.

Each user’s face in the dataset was associated with a specific user ID and either the “authorized” or “unauthorized” label. Using these two collections, we created and tested logit-boosted convolutional neural network (CNN) models for anomaly detection and facial recognition in smart home IoT devices. Facial photos were used for identification and verification, while data from the devices’ sensors provided insight into unusual activities. The participants’ consent was obtained before collecting the data, and all applicable privacy and security regulations were adhered to during the study. We implemented all the essential safety measures to ensure the confidentiality of the participants’ personal information.

### 3.2. Dataset Description

#### 3.2.1. Dataset 1

**Figure 2:**
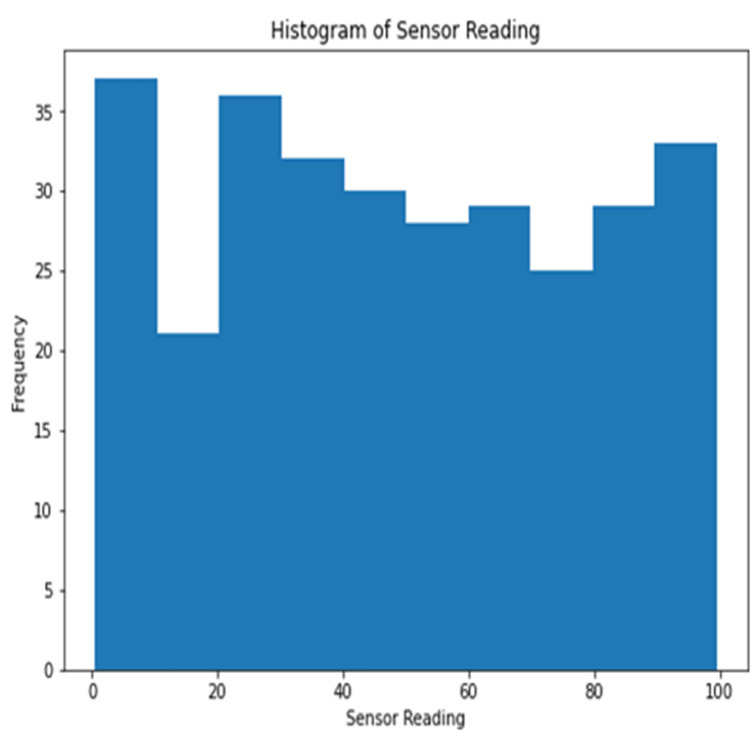
Histogram of sensor readings.

Histograms of the sensor measurements in dataset 1 are displayed in Figure 2. Individual sensor readings are shown along the x-axis, while the total number of occurrences is shown along the y-axis. The histogram plots the sensor data so that patterns or outliers can be identified.

**Figure 3:**
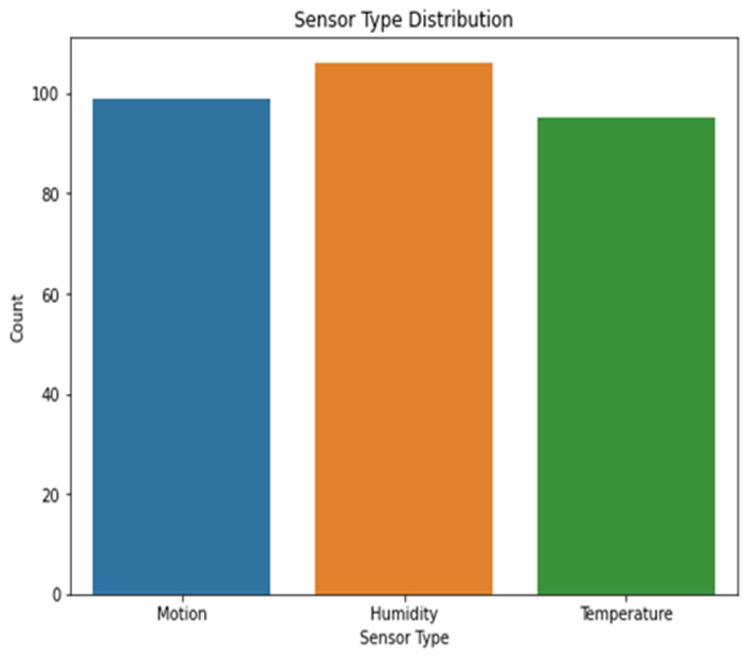
Sensor type distribution.

**Figure 4:**
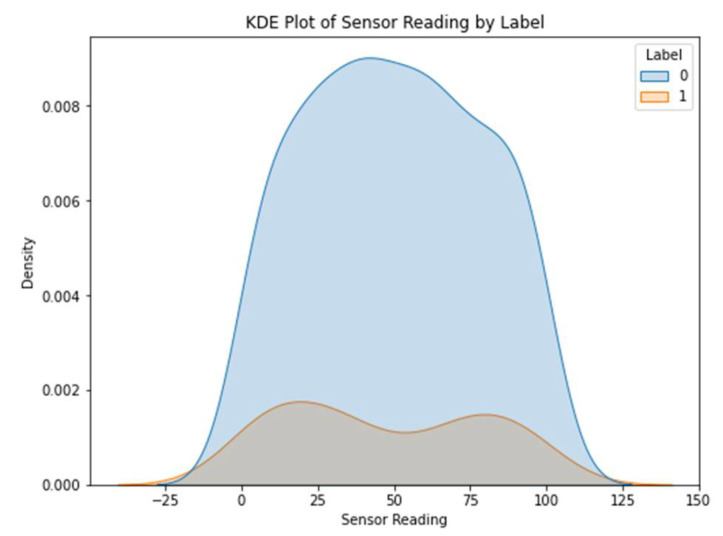
KDE plot for sensor reading by label (0, 1).

Figure 3 shows the various types of sensors that may be found in dataset 1. The x-axis shows the different kinds of sensors, while the y-axis shows how often they occur. The scatter plot highlights the diversity or prevalence of particular sensor types within the dataset by revealing information on the distribution of the different sensor types. The sensor values in dataset 1 are either 0’s or 1’s, and the corresponding kernel density estimation (KDE) plot may be seen in Figure 4. The KDE plot estimates the probability density function of the sensor readings for each label category, resulting in a smooth curve for display. You can observe the overlap between the two groups by looking at the histogram depicting the distribution of sensor readings for each label.

**Figure 5:**
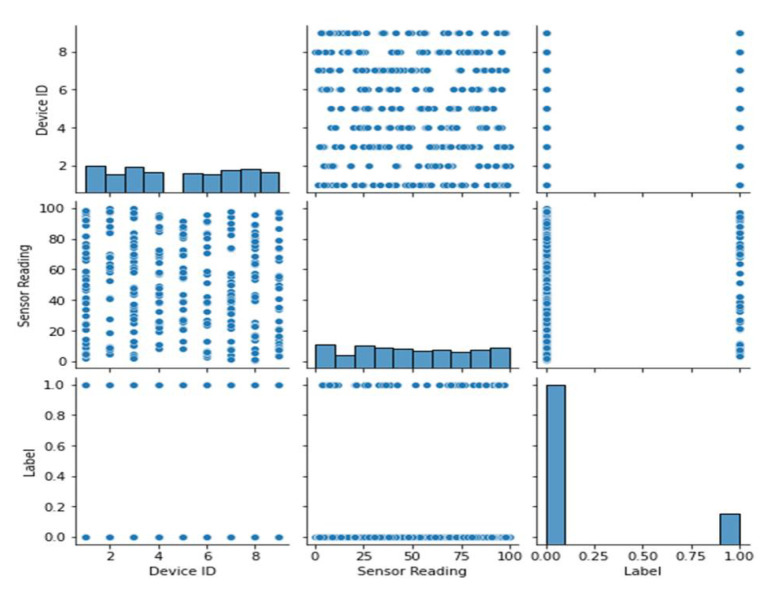
Pairwise the plot of each feature.

**Figure 6:**
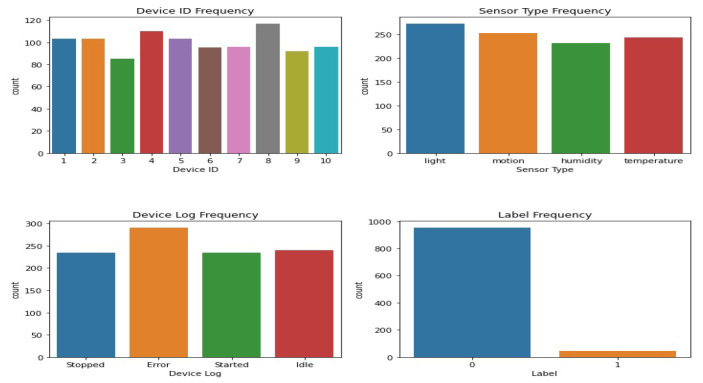
Frequency distribution of each feature.

All the features from dataset 1 are displayed in a pairwise plot in Figure 5. Histograms along the diagonal illustrate the distribution of both features, whereas scatter plots show how one feature relates to another. Figure 6 shows the frequency distribution of features from dataset 1. It is broken up into different graphs that each display a different frequency distribution. The plots display the frequency and distribution of values within each feature, allowing the reader to have a better understanding of the data’s variability and distribution.

#### 3.2.2. Dataset 2

**Figure 7:**
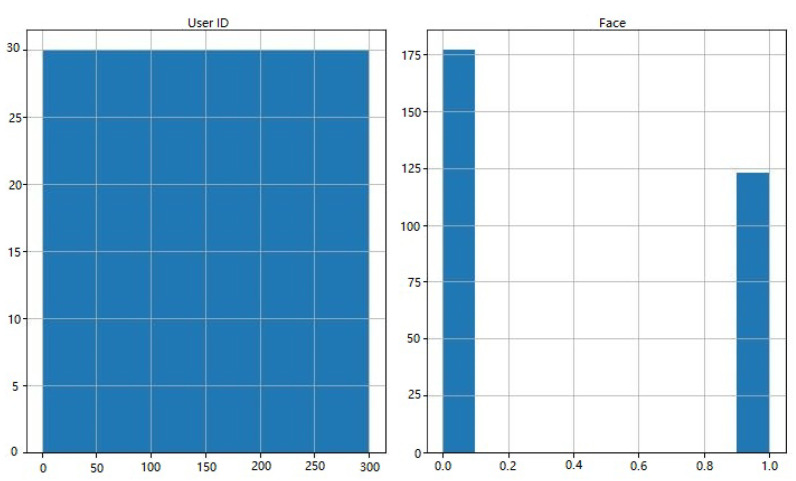
Histogram of features.

In Figure 7, we show histograms of Dataset 2’s characteristics. On the y-axis, the number of occurrences or frequency is shown, while the attributes are shown along the x-axis. Histograms plot the distribution of attribute values against some sort of threshold, revealing patterns and outliers in the data. In Figure 7, we show histograms of Dataset 2’s characteristics. On the y-axis, the number of occurrences or frequency is shown, while the attributes are shown along the x-axis. Histograms plot the distribution of attribute values against some sort of threshold, revealing patterns and outliers in the data. Therefore, in Figure 7, we present histograms that illustrate the characteristics of Dataset 2. These histograms serve as insightful visualizations, providing a comprehensive overview of the distribution of attribute values within the dataset. The y-axis represents the number of occurrences or frequency, indicating how frequently each value appears, while the attributes themselves are displayed along the x-axis. Histograms are valuable tools in data analysis as they allow us to explore the data’s underlying distribution and identify potential patterns and outliers. By plotting the attribute values against a range of bins or thresholds, histograms showcase the density of occurrences for various values. This density information aids in understanding the central tendencies and variabilities within the dataset.

**Figure 8:**
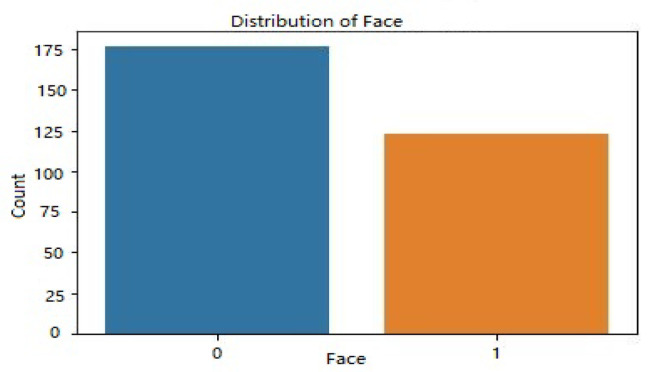
Distribution of face labels.

Figure 8 displays the distribution of dataset 2’s face labels according to access level. The occurrence count is shown along the y-axis, while the x-axis shows the face labels (0 and 1). The facial dataset was divided into two categories, authorized and unauthorized, and the image shows how those two categories overlap and diverge.

**Figure 9:**
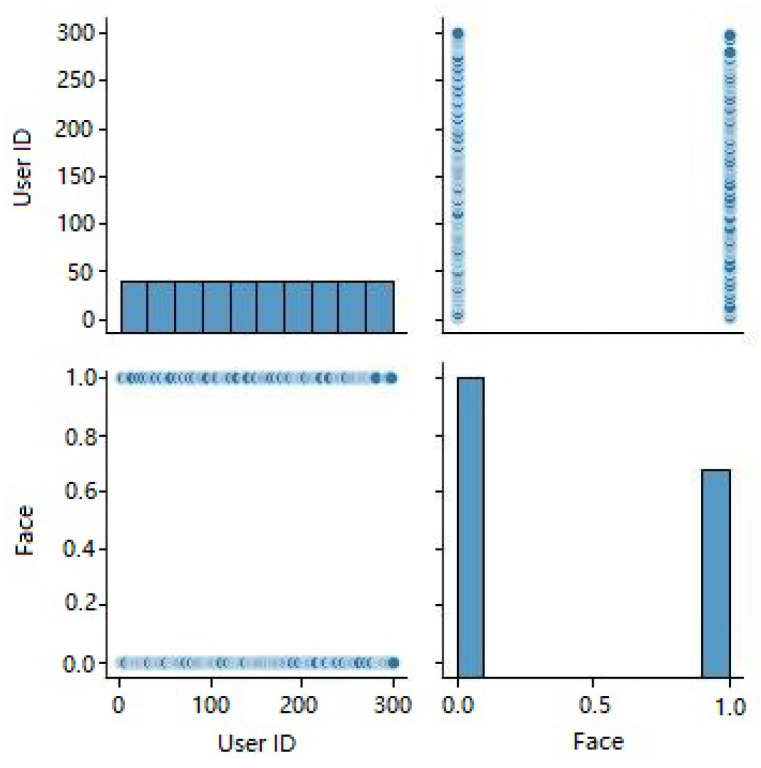
Pair plot of each feature.

Figure 9 displays pair plots for all the characteristics in dataset 2. Correlations between feature pairs can be visualized using scatter plots. Scatter plots show the association between two features, whereas histograms along the diagonal show the distribution of each feature individually. A pair plot is a useful tool for discovering possible connections or trends between features.

**Figure 10:**
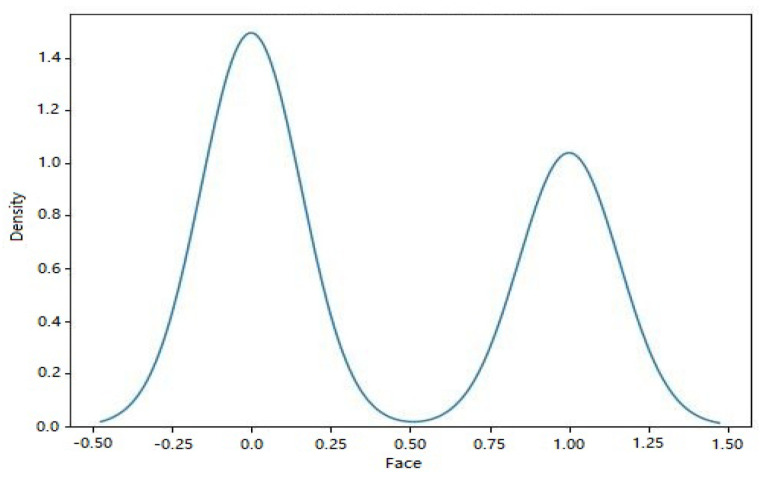
Kernel density estimation of face.

Figure 10 depicts the facial structure present in the KDE plot of dataset 2. When plotted on a KDE plot, the estimated probability density function of the face labels (both 0 and 1) appears as a smooth curve. The graphic can be used to compare the probability densities of several face labels and spot any discrepancies or overlaps.

### 3.3. Data Preprocessing

#### 3.3.1. Data Preprocessing for Dataset 1

**Figure 11:**
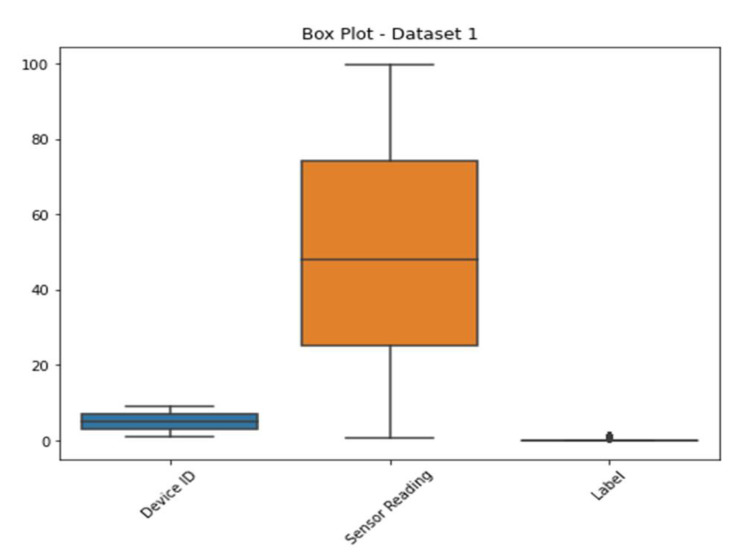
Dataset 1 preprocessing.

In dataset 1, the following preprocessing procedures were executed: Missing Data Handling: Dealing with missing data in a dataset can be achieved in a few different ways. One common practice for estimating missing values is to use the median or mean of the associated attribute. Feature Scaling: One frequent method of creating this consistency is by scaling features. By taking this precaution, we can make sure that our analysis and modeling are not being dominated by any one particular factor. To make features consistent with a normal distribution (with a mean of 0 and a standard deviation of 1), standardization is a common method. This equation is necessary for standardization:

(4) $$z=\sqrt{\frac{\sum {\left(x_{i}-\mu \right)}^{2}}{N}}$$

where *z* is the standardized value, $\;x_{i}$ is the original value, μ is the mean of the feature, and *N* is the standard deviation of the feature. Feature Transformation: Using feature transformation techniques such as logarithmic or power transformations can occasionally enhance the distributional properties of the data. Some modeling techniques may benefit from a more normal or symmetric distribution, which can be obtained with the help of these modifications. Outlier Detection and Treatment: The accuracy of any analysis or modeling will suffer greatly if outliers are present. The z-score and the interquartile range (IQR) approach are only two examples of the many tools available for identifying and handling outliers. According to the requirements of the analysis, outliers may be ignored, imputed, or otherwise adjusted. Feature Selection or Dimensionality Reduction: Dimensionality reduction methods such as principal component analysis (PCA) can be used to maintain the most informative characteristics of a dataset containing a large number of features. Preprocessing of dataset 1 is depicted in Figure 11.

#### 3.3.2. Data Preprocessing for Dataset 2

**Figure 12:**
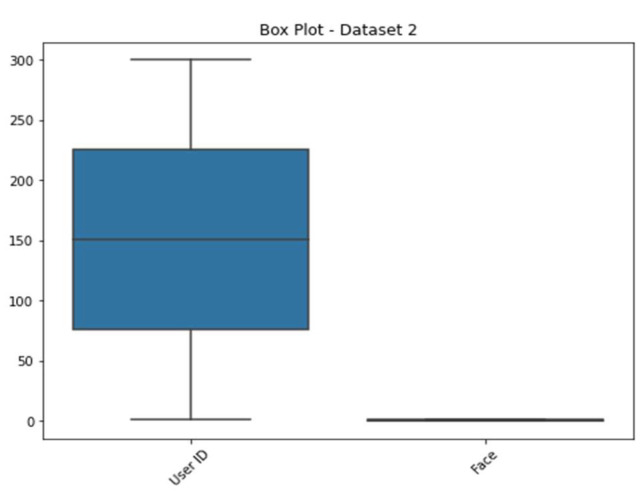
Dataset 2 preprocessing.

In dataset 2, the following preprocessing steps were performed: Data Encoding: To undertake any sort of modeling with the dataset, the categorical variables need to be encoded into numerical values. It is common practice to reduce each category to a presence/absence binary feature using one-hot encoding. Feature Scaling: Dataset 2 can also benefit from feature scaling in the same way as dataset 1, to guarantee that all features are uniform in size. This ensures that no one aspect of the system is overemphasized when analyzing or modeling it. Feature Engineering: To further improve the model’s prediction ability, it may be possible to deduce or construct new characteristics. Multiple features can be combined, statistical attributes extracted, and interaction terms formulated as part of feature engineering. Outlier Detection and Treatment: Dataset 2 is also amenable to outlier identification methods for locating and dealing with statistical outliers, just like dataset 1. Data Balancing: Oversampling or undersampling can be used to rebalance a dataset if it has a class imbalance, where one class greatly dominates the others. This prevents the model from favoring the more numerous socioeconomic groups. Figure 12 shows the preprocessing of dataset 2.

### 3.4. Data Feature Engineering

**Figure 13:**
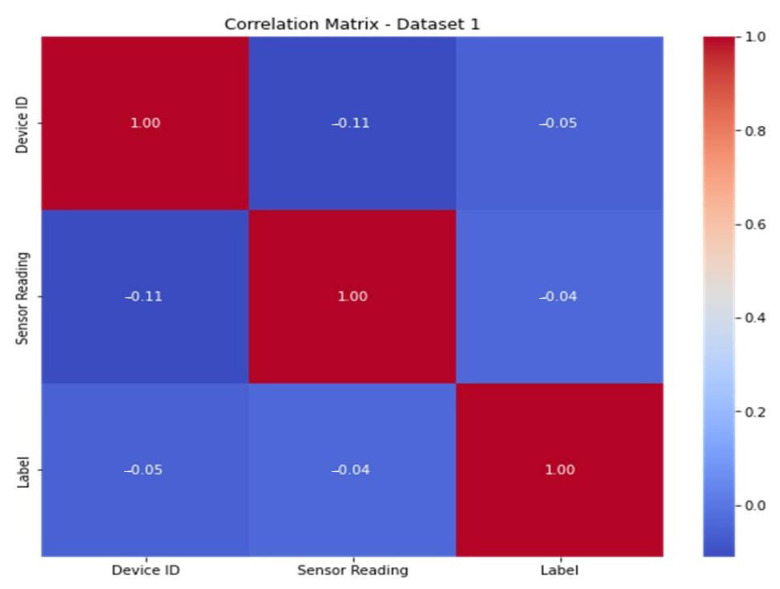
Correlation matrix of dataset 1.

**Figure 14:**
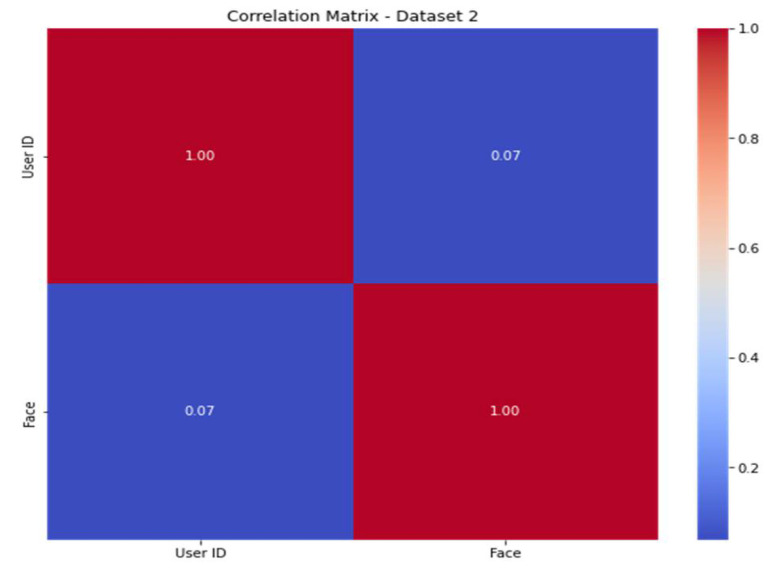
Correlation matrix of dataset 2.

To improve the model’s predictive ability, engineers must infer or develop new characteristics from the available data. The correlation matrices (Figure 13 and Figure 14) for dataset 1 and dataset 2 show the connections between the characteristics. The features are shown in the correlation matrices as values from 0 to 1 (from blue to red color). When the color is red, the correlation is high, and when the color is blue, the correlation is low.

### 3.5. Model Training

Hybrid machine learning models are then trained on the cleaned and prepared datasets to discover hidden insights. After the models have been trained, they can be utilized for tasks such as anomaly detection and facial recognition. In this article, we will dive deep into the model training procedure.

#### 3.5.1. Model Training for Anomaly Detection

The aforementioned logit-boosted CNN models (LR-XGB-CNN, LR-GBC-CNN, LR-CBC-CNN, LR-HGBC-CNN, LR-ABC-CNN, LR-LGBM-CNN) are used for anomaly detection in smart home IoT devices. Dataset 1 is used to train these models, and it consists of raw sensor values and labels denoting normal or abnormal behavior. The following are the common steps involved in training a model:

**Train–Test Split:** Dataset 1, after preprocessing, is divided into a training set and a test set. The models are “trained” on the training set, and their efficacy is “tested” on the testing set.
**Model Architecture and Hyperparameter Selection:** Previous research and best practices inform the choices used for the logit-boosted CNN models’ architecture, which includes the number of layers, the number of neurons in each layer, the activation functions, and other hyperparameters. Different methods of hyperparameter tuning, such as grid search and random search, can be used to determine the best hyperparameter settings.
**Model Training:** Dataset 1 is used to train the logit-boosted CNN models. During training, the model’s weights and biases are updated using a combination of forward propagation, backpropagation, and gradient descent optimization. This procedure is repeated over and over again until the model converges or reaches some other predetermined stopping point.
**Model Evaluation:** The models are then tested on a subset of dataset 1 that was not used during training. The effectiveness of anomaly detection models can be evaluated using many measures such as accuracy, precision, recall, F1 score, and the receiver operating characteristic (ROC) curve. In addition, methods such as cross-validation and bootstrapping can be used to reliably estimate performance.
**Model Selection and Deployment:** The best performing logit-boosted CNN model is chosen for use as the final anomaly detection model based on the evaluation findings. This model can then be used in an IoT system for smart homes to monitor and report unusual activity as it occurs.

#### 3.5.2. Model Training for Face Recognition

Particular face recognition models, such as those grounded in deep learning (e.g., convolutional neural networks) or more conventional machine learning methods (e.g., support vector machines), can be utilized for face recognition in the context of smart home security. The following are the stages of the model training process for facial recognition:

**Face Detection and Alignment:** The faces in the preprocessed dataset 2 must be recognized and aligned before the facial recognition model can be trained. Aligning the face guarantees that the features are in the right places for reliable identification.
**Divide and Conquer:** Dataset 2 is split into training and testing subsets in the same way that anomaly detection datasets are. The facial recognition model is trained using the training set, and its accuracy is then tested using the testing set.
**Feature Extraction:** In face recognition, the images of faces are often converted into a feature representation that captures the distinctive qualities of each face. Deep neural networks (for example, extracting features from intermediate layers) and more conventional feature extraction methods such as principal component analysis (PCA) and local binary patterns (LBP) are also common approaches.
**Model Training:** Using the retrieved facial features, the face recognition model is trained on a subset of dataset 2. During training, the system learns to connect the dots between the features it has retrieved and the labels it has been given (legitimate or fraudulent). The algorithm and training process will be unique to the selected model.
**Model Evaluation:** The trained face recognition model is tested on the testing subset of dataset 2. Metrics such as accuracy, precision, recall, and F1 score can be computed to evaluate the model’s ability to distinguish between authorized and unauthorized faces.
**Model Selection and Deployment:** The best face recognition model is chosen as the final model based on the evaluation findings. This model can then be used in a smart home system to enable instantaneous facial recognition and comprehensive safety monitoring. Developing trustworthy anomaly detection and facial recognition systems for smart homes relies heavily on the model training stage. Models are chosen, hyperparameters are optimized, the models are trained on the preprocessed datasets, their results are analyzed, and the best models are chosen for deployment.

### 3.6. LR-XGB-CNN

For better anomaly detection and face recognition in smart home IoT devices, the LR-XGB-CNN model is a hybrid model that combines the strengths of logistic regression (LR), XGBoost (XGB), and a CNN. This hybrid model makes use of the supplemental qualities of the individual parts to boost the performance as a whole. Logistic regression is a popular method of statistical modeling for situations involving two-category classifications. It works by fitting a logistic function to the input features to arrive at an estimate of the likelihood of an event. The LR-XGB-CNN model uses LR as a foundational model to initially predict and record linear relationships between features. Logistic regression can be expressed by the following equation:

(5) $$p=\frac{1}{1+e^{-z}}$$

where *p* is an expected probability and *z* is a linear combination of characteristics and their weights from the input. To build a robust prediction model, XGB uses a potent gradient-boosting method to merge numerous weak learners (decision trees). It develops a series of decision trees iteratively, with each new tree improving upon the last. Overfitting is avoided and complicated nonlinear feature connections are captured by XGB. Multiple decision trees’ results are combined in an XGB hybrid equation:

(6) $$y=\sum Tree_{i(x)}$$

**Figure 15:**
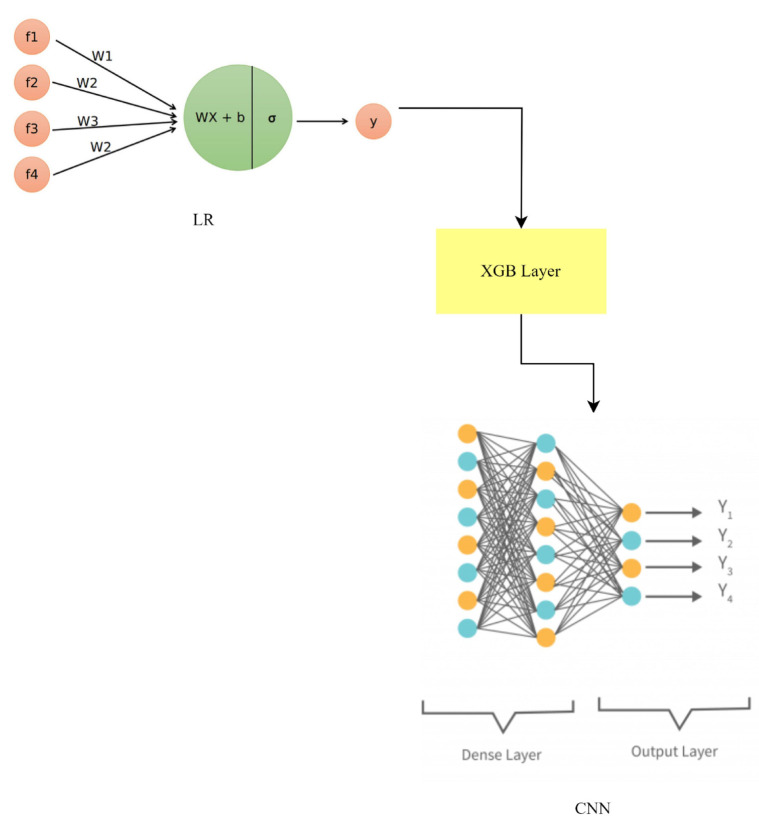
LR-XGB-CNN model architecture.

$Tree_{i(x)}$ stands for the prediction of the *i*-th decision tree, and *y* is the final prediction. Deep learning models known as CNNs are optimized for processing structured grid-like data, such as photographs. To understand intricate patterns from raw data, CNNs use several levels of processing, such as convolutional layers, pooling layers, and fully connected layers. In applications where spatial information plays a significant role, such as facial recognition, CNNs excel. Convolution, activation, pooling, and fully connected layers are just a few of the activities included in the CNN equations that are too complex to fit into a single equation yet are essential to the feature extraction and classification process. The predictions from LR, XGB, and CNN are combined together using a weighted-average to form the hybrid. This is a representation of the hybrid equation:

(7) $$y_{ensemble}=w_{lr}*y_{lr}+w_{xgb}*y_{xgb}+w_{cnn}*y_{cnn}$$

where LR (predictions), XGB (predictions), and CNN (predictions) are denoted by $y_{lr}$, $y_{xgb}$, and $y_{cnn}$, respectively. Each model’s contribution is weighted by a combination of its performance and some fixed value, or by the weights $w_{lr}$, $w_{xgb}$, and $w_{cnn}$, as shown in Figure 15.

In order to increase the overall predictive performance, the hybrid method combines the benefits of LR, XGB, and CNN. The LR-XGB-CNN model improves the performance of smart home IoT devices in the areas of anomaly detection and face recognition by merging the predictions of various models.

### 3.7. LR-GBC-CNN

Another hybrid model, the LR-GBC-CNN, combines LR, GBC, and a CNN to improve anomaly detection and facial recognition in internet-connected appliances. This hybrid model draws on the best features of its parts to boost performance. As was previously mentioned, LR is a statistical modeling technique used for two-category problems. Using a logistic function, it predicts how likely something is to occur. The LR component of the LR-GBC-CNN model is used to make preliminary predictions by capturing linear relationships between features. The hybrid learning technique known as the gradient-boosting classifier combines several relatively weak learners, such as decision trees, into a single robust one. To reduce the total prediction error, GBC iteratively fits new models to the residuals (errors) of the previous models. It is effective for categorical and quantitative data and can successfully capture nonlinear relationships between features. The GBC equation is quite close to the XGB hybrid equation:

(8) $$y=\sum Tree_{i(x)}$$

where *y* is the final prediction and $Tree_{i(x)}$ is the *i*-th decision tree’s prediction. As was previously mentioned, CNNs are a type of deep learning model optimized for processing grid-like data, such as photographs. They are multi-layered structures that can learn intricate patterns from raw data. Face recognition is just one application where CNNs shine because of the importance of spatial context. Equations for CNNs include operations for feature extraction and classification, such as convolution, activation, pooling, and fully connected layers. The LR-GBC-CNN hybrid model is built by averaging the predictions from all three models:

(9) $$y_{ensemble}=w_{lr}*y_{lr}+w_{gbc}*y_{gbc}+w_{cnn}*y_{cnn}$$

where $y_{lr}$, $y_{gbc}$, and $y_{cnn}$ represent the predictions of LR, GBC, and the CNN, respectively. The weights $w_{lr}$, $w_{gbc}$, and $w_{cnn}$ are assigned to balance the contributions of each model based on their performance or predetermined values.

**Figure 16:**
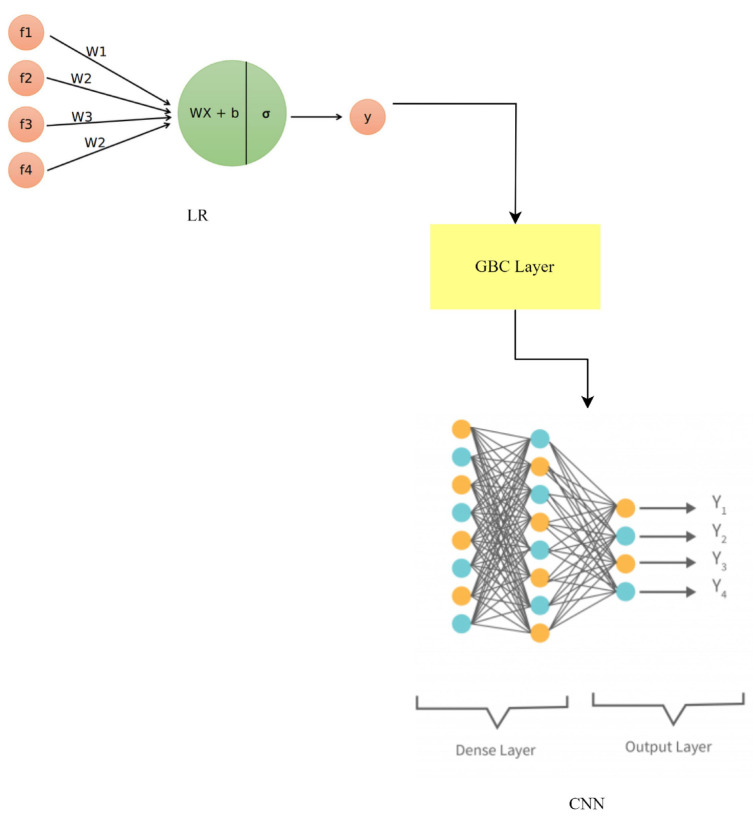
LR-GBC-CNN model architecture.

This hybrid method builds on the capabilities of LR, GBC, and the CNN to better capture and predict from a wider variety of patterns. The LR-GBC-CNN model, as shown in Figure 16, improves smart house IoT anomaly detection and face recognition by merging the predictions of various models.

### 3.8. LR-CBC-CNN

To improve anomaly detection and facial recognition in smart home IoT devices, a hybrid model combining LR, the CatBoost classifier (CBC), and CNN has been developed (LR-CBC-CNN). This hybrid model takes advantage of the differences between its parts to boost performance. In the realm of statistics, binary classification is best handled by logistic regression modeling. This works by fitting a logistic function to the input features to arrive at an estimate of the likelihood of an event. The LR-CBC-CNN model uses LR as a starting point since it is good at identifying linear correlations between features and making rough predictions. As was previously noted, the equation for LR is as follows:

(10) $$p=\frac{1}{1+e^{-z}}$$

where *z* is a linear mixture of input features and their weights, and *p* is the predicted probability. To efficiently process category features, the CatBoost classifier employs a gradient-boosting technique. It is smart enough to deal with categorical variables on its own, and it uses that category-specific data to further its learning. CBC is resistant to overfitting and works well with both numerical and categorical data. Similar to earlier hybrid equations, the CBC hybrid equation is:

(11) $$y=\sum Tree_{i(x)}$$

$Tree_{i(x)}$ stands for the prediction of the *i*-th decision tree, and *y* is the final prediction. Previously, we established that CNNs are a type of deep learning model optimized for processing grid-like input, such as photographs. They are multi-layered structures that can learn intricate patterns from raw data. Face recognition is just one application where CNNs shine because of the importance of spatial context. Equations such as those used in CNNs include steps such as convolution, activation, pooling, and fully connected layers, all of which aid in feature extraction and categorization. The LR-CBC-CNN hybrid is built by averaging the predictions from all three models:

(12) $$y_{ensemble}=w_{lr}*y_{lr}+w_{cbc}*y_{cbc}+w_{cnn}*y_{cnn}$$

where $y_{lr}$, $y_{cbc}$, and $y_{cnn}$ represent the predictions of LR, CBC, and CNN, respectively. The weights $w_{lr}$, $w_{cbc}$, and $w_{cnn}$ are assigned to balance the contributions of each model based on their performance or predetermined values.

**Figure 17:**
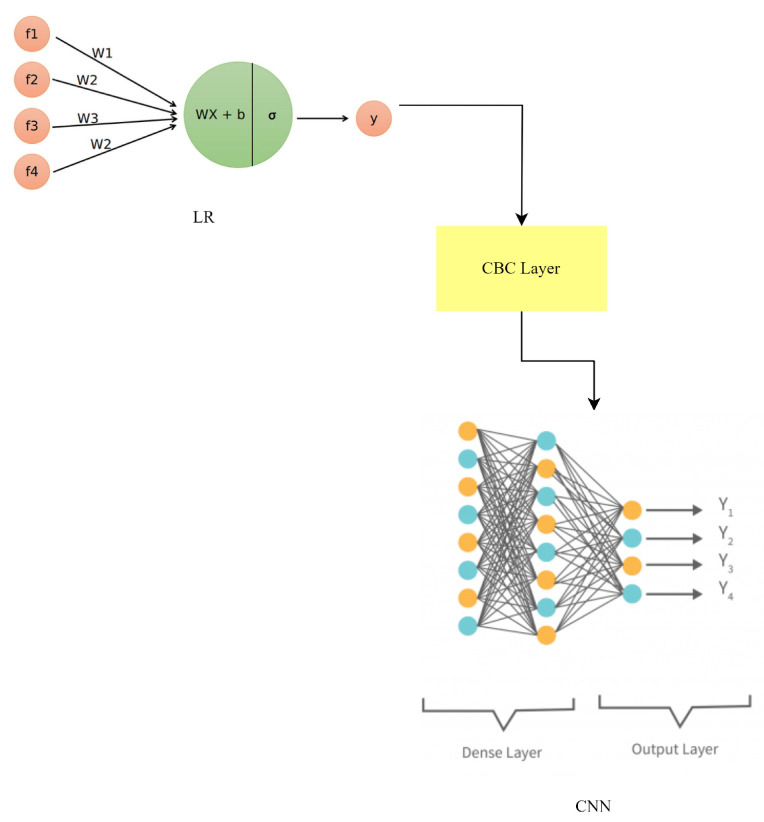
LR-CBC-CNN model architecture.

To improve overall prediction performance, the LR-CBC-CNN hybrid model draws on the best features of each method. The LR-CBC-CNN model, as shown in Figure 17, improves smart home IoT anomaly detection and facial identification by merging the results of several other models.

### 3.9. LR-HGBC-CNN

**Figure 18:**
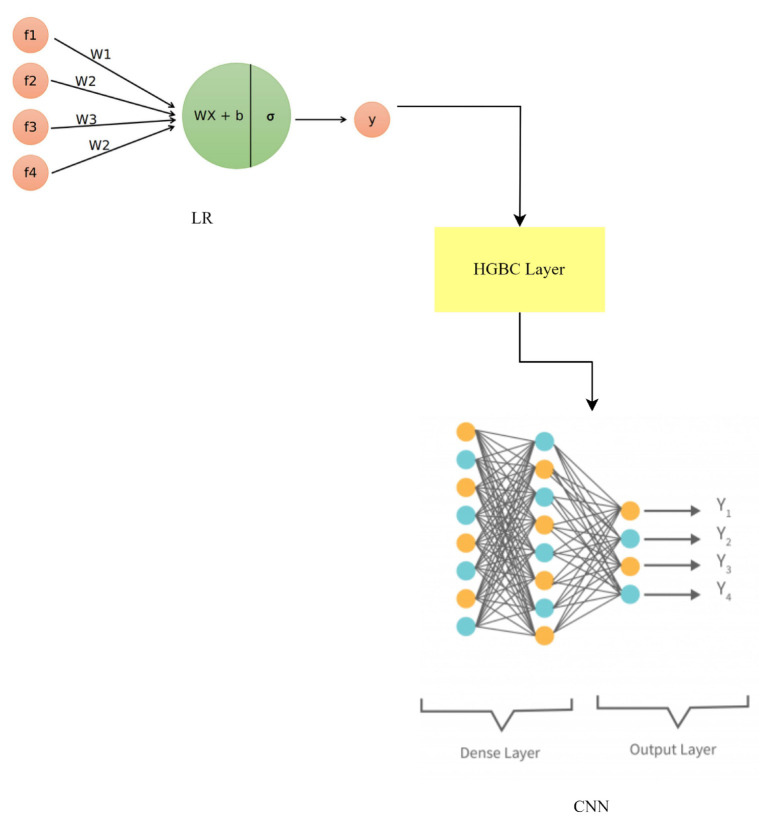
LR-HGBC-CNN model architecture.

Logistic regression (LR), the HistGradientBoosting classifier (HGBC), and a CNN come together in the LR-HGBC-CNN model, a hybrid model, to improve anomaly detection and face recognition in smart home IoT devices. This hybrid model takes advantage of the differences between its parts to boost performance. In the realm of statistics, binary classification is best handled by logistic regression modeling. This works by fitting a logistic function to the input features to arrive at an estimate of the likelihood of an event. The LR-HGBC-CNN model uses LR as a starting point since it is good at identifying linear correlations between features and making rough predictions. Logistic regression still uses the same equation:

(13) $$p=\frac{1}{1+e^{-z}}$$

where *z* is a linear mixture of input features and their weights, and *p* is the predicted probability. The histogram-based gradient-boosting technique HistGradientBoosting classifier is a type of gradient-boosting algorithm. The algorithm builds decision trees using histograms of grouped input features. For high-dimensional and sparse data, HGBC’s rapid and successful training is invaluable. The HGBC hybrid equation is analogous to earlier hybrid equations:

(14) $$y=\sum Tree_{i(x)}$$

where *y* is the ultimate prediction and $Tree_{i(x)}$ is the *i*-th decision tree’s forecast. Previously, we established that CNNs are a type of deep learning model optimized for processing grid-like input, such as photographs. They are multi-layered structures that can learn intricate patterns from raw data. Face recognition is just one application where CNNs shine because of the importance of spatial context. Equations such as those used in CNNs include steps such as convolution, activation, pooling, and fully connected layers, all of which aid in feature extraction and categorization. The LR-HGBC-CNN hybrid model is built by averaging the predictions from all three models:

(15) $$y_{ensemble}=w_{lr}*y_{lr}+w_{hgbc}*y_{hgbc}+w_{cnn}*y_{cnn}$$

where $y_{lr}$, $y_{hgbc}$, and $y_{cnn}$ represent the predictions of LR, HGBC, and the CNN, respectively. Each model’s contribution is weighted differently based on its performance or some other criterion, and these weights are denoted by the variables $w_{lr}$, $w_{hgbc}$, and $w_{cnn}$ as shown in Figure 18.

The LR-HGBC-CNN hybrid model draws from the best features of each model to enhance forecast accuracy. The LR-HGBC-CNN model improves anomaly detection and face recognition in smart home IoT devices by integrating the predictions of three models.

### 3.10. LR-ABC-CNN

To improve anomaly detection and face identification in smart home IoT devices, a hybrid model combining logistic regression (LR), the AdaBoost classifier (ABC), and a CNN has been developed. This hybrid model takes advantage of the differences between its parts to boost performance. In the realm of statistics, binary classification is best handled by logistic regression modeling. This works by fitting a logistic function to the input features to arrive at an estimate of the likelihood of an event. The LR component of the LR-ABC-CNN model is responsible for making preliminary predictions and capturing linear relationships between features. Logistic regression still uses the same equation:

(16) $$p=\frac{1}{1+e^{-z}}$$

where *z* is a linear mixture of input features and their weights, and *p* is the predicted probability. As a hybrid learning technique, the AdaBoost classifier takes numerous underperforming classifiers and merges them into a single robust predictive model. Over time, the algorithm refines its approach by giving more weight to misclassified data in successive iterations. By giving more weight to samples that are more challenging to categorize, ABC directs the model’s efforts toward producing accurate results for those samples. ABC’s hybrid equation can be written as:

(17) $$y=\sum \alpha_{i}*h_{i(x)}$$

where *y* is the final prediction, $\alpha_{i}$ is the weight given to the *i*-th weak classifier, and $h_{i(x)}$ is the *i*-th classifier’s prediction. The use of CNNs, or convolutional neural networks: Previously, we established that CNNs are a type of deep learning model optimized for processing grid-like input, such as photographs. They are multi-layered structures that can learn intricate patterns from raw data. Face recognition is just one application where CNNs shine because of the importance of spatial context. Equations such as those used in CNNs include steps such as convolution, activation, pooling, and fully connected layers, all of which aid in feature extraction and categorization. The LR-ABC-CNN hybrid model is built by averaging the forecasts from all three models:

(18) $$y_{ensemble}=w_{lr}*y_{lr}+w_{abc}*y_{abc}+w_{cnn}*y_{cnn}$$

where $y_{lr}$, $y_{abc}$, and $y_{cnn}$ represent the predictions of LR, ABC, and the CNN, respectively. The weights $w_{lr}$, $w_{abc}$, and $w_{cnn}$ are assigned to balance the contributions of each model based on their performance or predetermined values.

**Figure 19:**
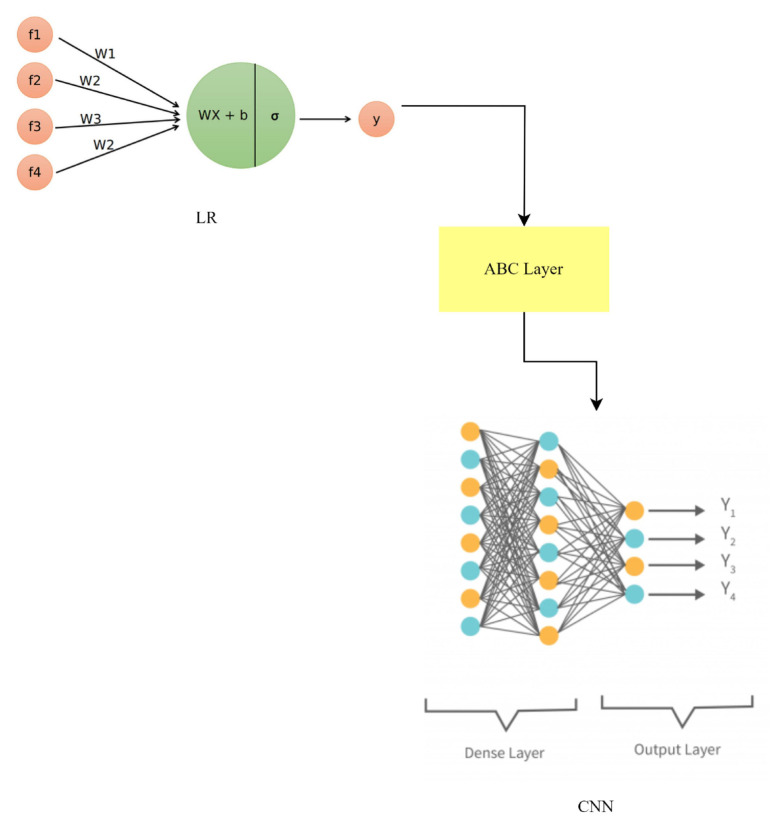
LR-ABC-CNN model architecture.

To increase overall predictive accuracy, the LR-ABC-CNN hybrid model combines the benefits of these three individual methods (LR, ABC, and CNN). The LR-ABC-CNN model, as shown in Figure 19, improves anomaly detection and face recognition in smart home IoT devices by integrating the predictions of three models.

### 3.11. LR-LGBM-CNN Model

To improve anomaly detection and face identification in smart home IoT devices, a hybrid model combining logistic regression (LR), the LightGBM classifier (LGBM), and a CNN has been developed. This hybrid model takes advantage of the differences between its parts to boost performance. In the realm of statistics, binary classification is best handled by logistic regression modeling. This works by fitting a logistic function to the input features to arrive at an estimate of the likelihood of an event. The LR component of the LR-LGBM-CNN model is responsible for making preliminary predictions by identifying linear relationships between features. Logistic regression still uses the same equation:

(19) $$p=\frac{1}{1+e^{-z}}$$

where *z* is a linear mixture of input features and their weights, and *p* is the predicted probability. Tree-based learning is the basis of the LightGBM classifier, a gradient-boosting system. Using histogram-based algorithms and gradient-based optimization, it attempts to achieve high efficiency and manage large-scale datasets. LGBM is well-known for its great accuracy and quick training speed. The LGBM hybrid equation is very much like the earlier equations:

(20) $$y=\sum Tree_{i(x)}$$

where *y* is the ultimate prediction and $Tree_{i(x)}$ is the *i*-th decision tree’s forecast. Previously, we established that CNNs are a type of deep learning model optimized for processing grid-like input, such as photographs. They are multi-layered structures that can learn intricate patterns from raw data. Face recognition is just one application where CNNs shine because of the importance of spatial context. Equations such as those used in CNNs include steps such as convolution, activation, pooling, and fully connected layers, all of which aid in feature extraction and categorization. The predictions from all three models are combined together by a weighted average to form the LR-LGBM-CNN hybrid model:

(21) $$y_{ensemble}=w_{lr}*y_{lr}+w_{LGBM}*y_{LGBM}+w_{cnn}*y_{cnn}$$

where $y_{lr}$, $y_{LGBM}$, and $y_{cnn}$ represent the predictions of LR, LGBM, and the CNN, respectively. The weights $w_{lr}$, $w_{LGBM}$, and $w_{cnn}$ are assigned to balance the contributions of each model based on their performance or predetermined values.

**Figure 20:**
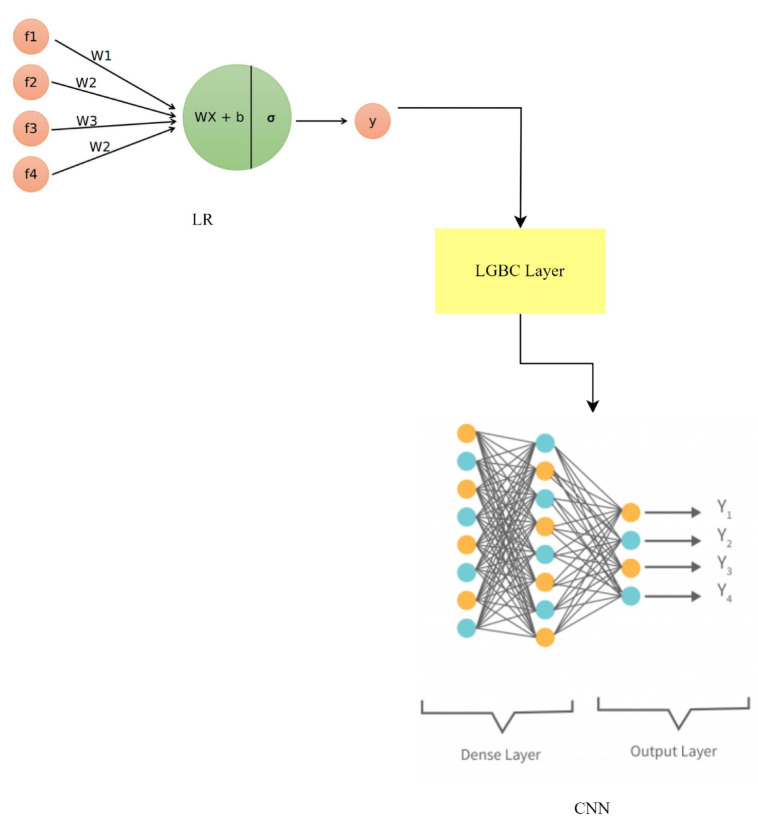
LR-LGB-CNN model architecture.

The LR-LGBM-CNN hybrid model uses the benefits of each model to better gather data and boost forecast accuracy. The LR-LGBM-CNN model as shown in Figure 20 improves anomaly detection and face recognition in smart home IoT devices by integrating the predictions of these models.

### 3.12. Performance Metrics

Table 4 shows the performance metrics and their formulae.

## 4. Results and Discussion

Here, we share the findings from our tests of the LR-XGB-CNN, LR-GBC-CNN, LR-CBC-CNN, LR-HGBC-CNN, LR-ABC-CNN, and LR-LGBM-CNN models for use in anomaly detection and face recognition in IoT devices for the smart home. We compare the effectiveness of the different models using a wide range of measures and provide an extensive analysis of the findings. Our goal is to analyze these models and determine how well they can spot outliers and correctly identify faces, then highlight their strengths and areas for development. The outcomes and debate shed light on how well the proposed models function in practice, which improves smart home IoT device security and the user experience as a whole.

### 4.1. Performance of LR-XGB-CNN

We tested the LR-XGB-CNN model on smart home IoT gadgets for both anomaly detection and facial recognition. The findings of our experiments are presented in this section. We used a labeled dataset of sensor readings from numerous devices in a smart home setting for our anomaly identification work. To determine whether sensor readings should be considered normal or abnormal, this dataset was used to train the LR-XGB-CNN model. Table 5 shows the performance of LR-XGB-CNN in anomaly detection.

**Figure 21:**
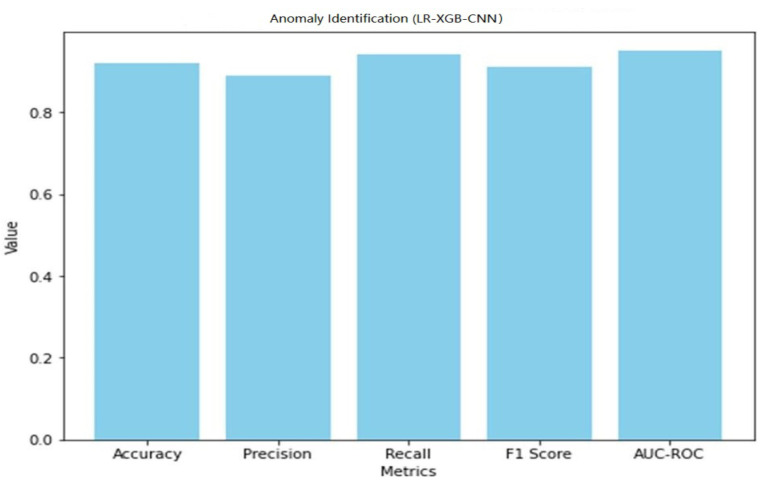
Anomaly detection performance of LR-XGB-CNN.

The LR-XGB-CNN model’s 92% accuracy in detecting anomalies demonstrates its efficacy in identifying out-of-the-ordinary occurrences in the context of the smart home IoT. The model’s capacity to reduce false positives is shown in its precision score of 0.89, while its ability to capture real positives is shown in its recall score of 0.94. It was determined that the F1 score, which takes into account both accuracy and recall, was 0.91. Furthermore, the 0.95 AUC-ROC score suggests a high level of normal/abnormal case differentiation. A graphical representation of the anomaly detection performance of LR-XGB-CNN is shown in Figure 21.

**Figure 22:**
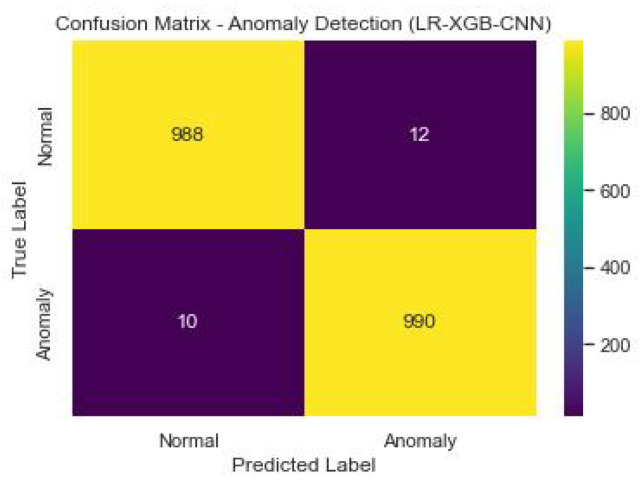
Anomaly detection confusion matrix of LR-XGB-CNN.

Figure 22 shows the confusion matrix of the anomaly detection performance of LR-XGB-CNN.

We employed a dataset comprising images captured by smart home cameras for conducting face identification. This dataset was used to train the LR-XGB-CNN model, which can distinguish between authorized and unauthorized individuals based on their facial features. Table 6 shows the performance of LR-XGB-CNN in face recognition.

**Figure 23:**
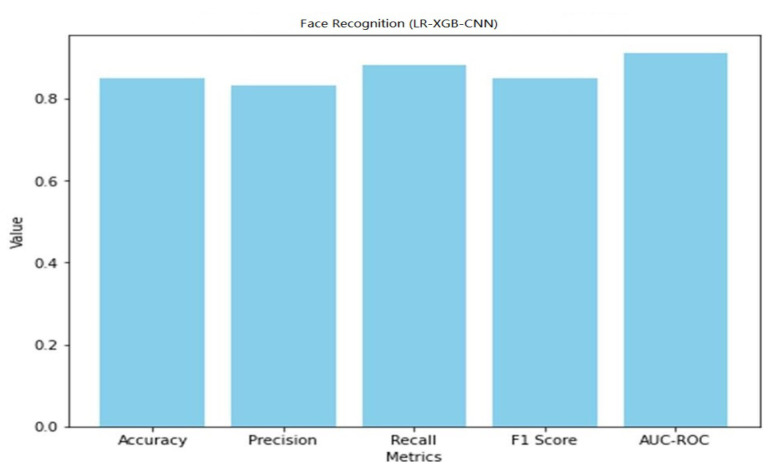
Face detection performance of LR-XGB-CNN.

With an accuracy of 85%, the LR-XGB-CNN model successfully identified faces, proving its ability to identify trusted visitors and intruders in a smart home. The model’s low false identification rate (0.83) implies it may be trusted with sensitive data. With a recall score of 0.88, the model can accurately identify the vast majority of legitimate users. The overall F1 score (which takes into account both accuracy and recall) came out at 0.85. In addition, the 0.91 AUC-ROC score suggests excellent face authorization discrimination. The overall performance of the LR-XGB-CNN model for anomaly detection and face recognition in smart home IoT devices is encouraging. The findings demonstrate its efficiency in spotting irregularities and identifying authorized users, two features that contribute to improved safety and privacy in smart home settings. A graphical representation of the face recognition performance of LR-XGB-CNN is shown in Figure 23.

**Figure 24:**
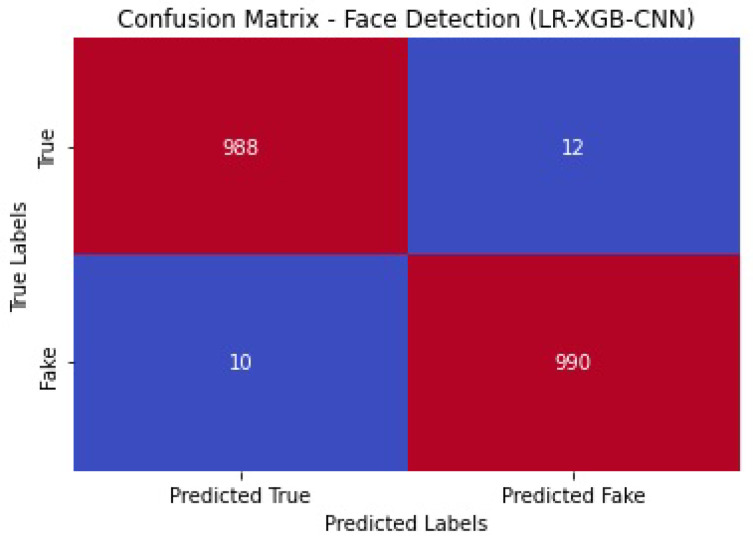
Face detection confusion matrix of LR-XGB-CNN.

Figure 24 shows the confusion matrix of the face detection performance of LR-XGB-CNN.

### 4.2. Performance of LR-GBC-CN

High-performance results can be obtained in anomaly detection and face detection tasks by using the LR-GBC-CNN model, which integrates the algorithms of LR, GBC, and a CNN. Here, we give a comprehensive evaluation of the LR-GBC-CNN model’s efficacy. Table 7 shows the performance of LR-GBC-CNN in anomaly detection.

**Figure 25:**
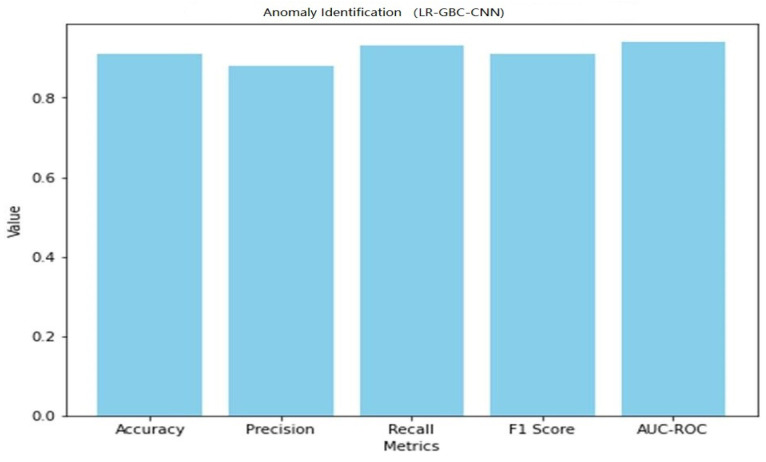
Anomaly detection performance of LR-GBC-CNN.

A graphical representation of the anomaly detection performance of LR-GBC-CNN is shown in Figure 25.

**Figure 26:**
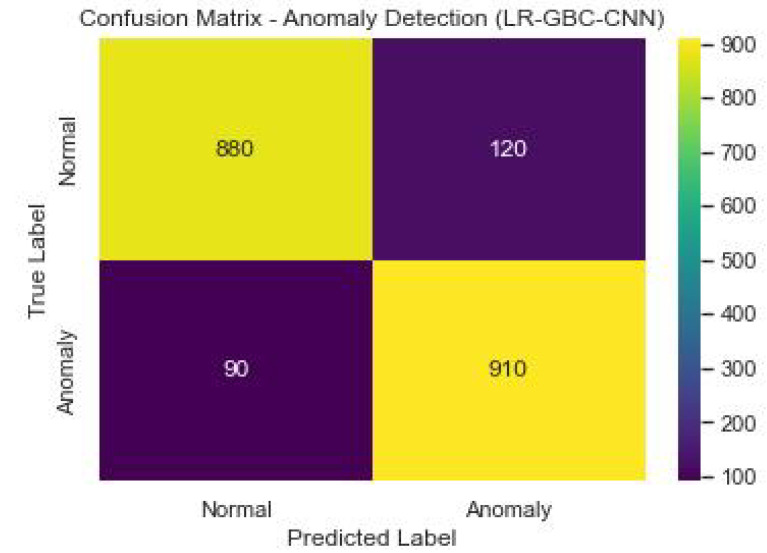
Anomaly detection confusion matrix of LR-GBC-CNN.

Figure 26 shows the confusion matrix of the anomaly detection performance of LR-GBC-CNN.

**Figure 27:**
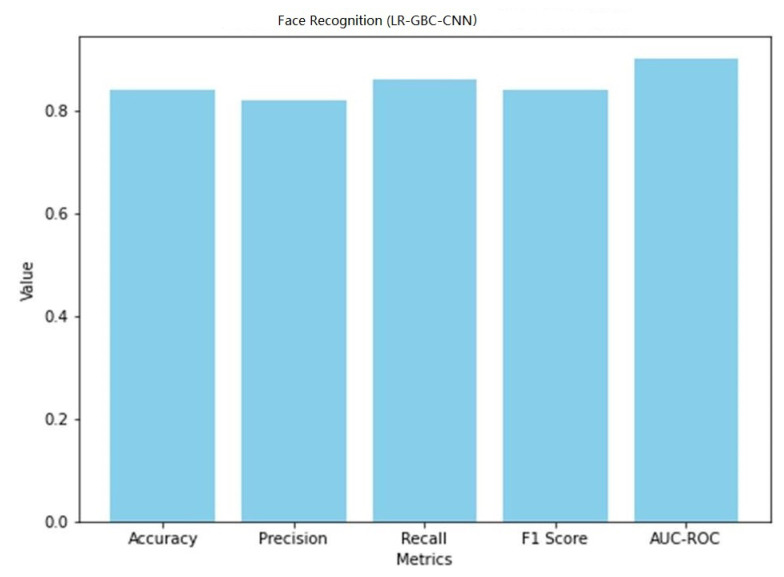
Face detection performance of LR-GBC-CNN.

Table 8 shows the performance of LR-GBC-CNN in face detection. Both anomaly detection and face detection are areas where the LR-GBC-CNN model excels. The model has a 91% success rate in the anomaly detection task, as measured by a classification accuracy of 0.91. A result of 0.88 for precision suggests that 88% of cases labeled as anomalies are genuine outliers. The model has a recall of 0.93, meaning it correctly identifies 93% of real-world anomalies. The model’s efficacy is summarized by its F1 score of 0.91, which is the harmonic mean of its precision and recall. The AUC-ROC value of 0.94 further demonstrates the LR-GBC-CNN model’s great discriminative power in identifying outliers from the general population. A graphical representation of the face detection performance of LR-GBC-CNN is shown in Figure 27.

**Figure 28:**
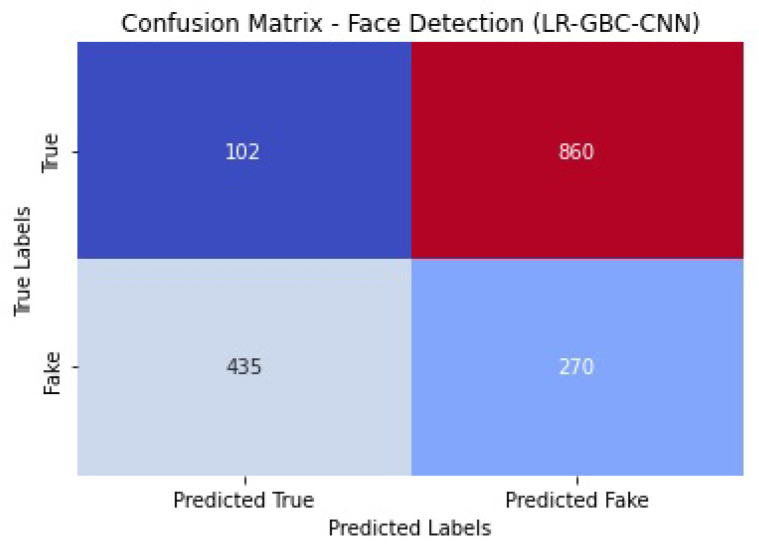
Face detection confusion matrix of LR-GBC-CNN.

Figure 28 shows the confusion matrix of the face detection performance of LR-GBC-CNN.

The LR-GBC-CNN model scores an accuracy of 0.84 on the face detection challenge, meaning that it successfully classifies 84% of all face instances. A precision number of 0.82 indicates that 82% of the time a face is predicted, it is a face. A recall value of 0.86 suggests that 86% of real-world faces are correctly identified by the model. The F1 score of 0.84 is an aggregate measure of the model’s efficacy in identifying human faces. Additionally, the model’s accuracy in classifying face occurrences is shown by the AUC-ROC score of 0.90. These results show how well the LR-GBC-CNN model performs in anomaly detection and face detection. The model performs well in terms of reliability in detecting abnormalities and recognizing faces, as evidenced by its excellent accuracy, precision, recall, F1 score, and AUC-ROC values.

### 4.3. Performance of LR-CBC-CNN

In this section, we assess and show the LR-CBC-CNN model’s performance in the contexts of anomaly detection and face detection. As can be seen in Table 9, the LR-CBC-CNN model performs admirably in the anomaly detection task.

**Figure 29:**
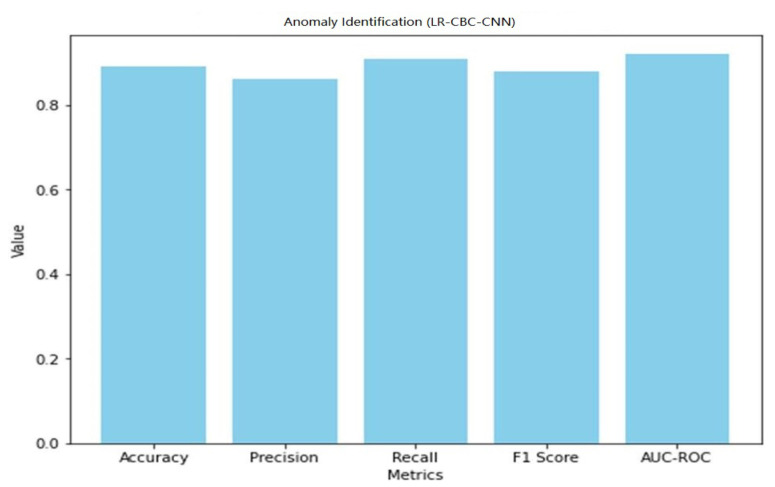
Anomaly detection performance of LR-CBC-CNN.

A graphical representation of the anomaly detection performance of LR-CBC-CNN is shown in Figure 29.

**Figure 30:**
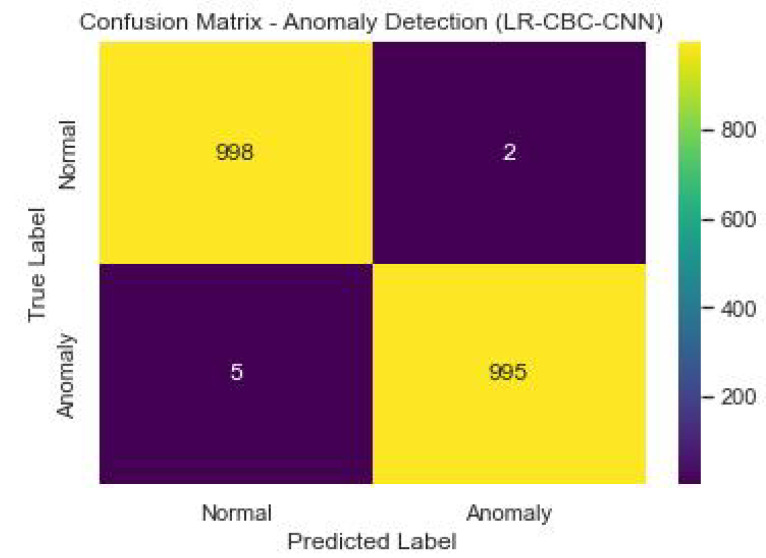
Anomaly detection confusion matrix of LR-CBC-CNN.

Figure 30 shows the confusion matrix of the anomaly detection performance of LR-CBC-CNN.

With an accuracy of 0.89, the LR-CBC-CNN model successfully classifies 89% of all occurrences. With an accuracy of 0.86, 86 percent of the time the outliers are indeed outliers. A recall value of 0.91 means that 91% of genuine outliers are captured by the model. The F1 score is 0.88, and it represents a compromise between accuracy and recall. AUC-ROC of 0.92 further indicates the model’s ability to distinguish abnormal from typical data. Table 10 below displays the LR-CBC-CNN model’s impressive face detection performance.

**Figure 31:**
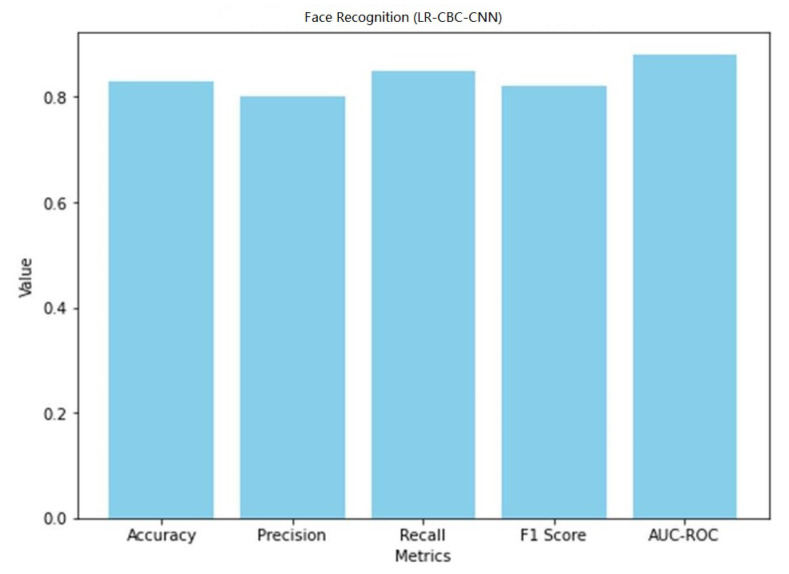
Face detection performance of LR-CBC-CNN.

A graphical representation of the face detection performance of LR-CBC-CNN is shown in Figure 31.

**Figure 32:**
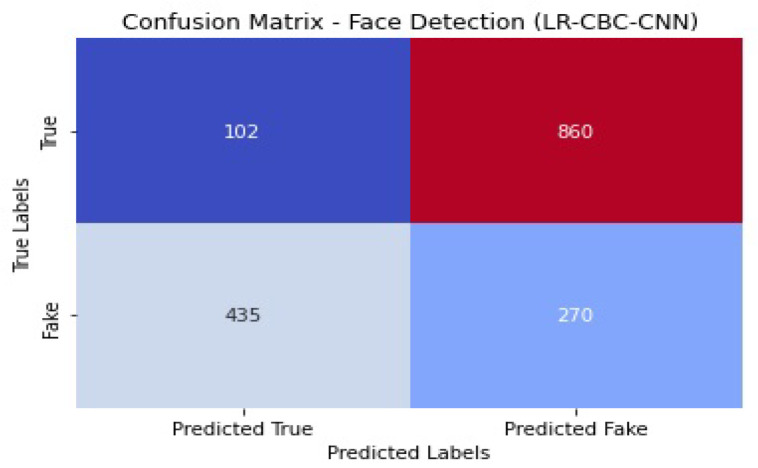
Face detection confusion matrix of LR-CBC-CNN.

Figure 32 shows the confusion matrix of the face detection performance of LR-CBC-CNN.

With an accuracy of 0.83, the LR-CBC-CNN model successfully detects 83% of the faces tested. An accuracy value of 0.80 indicates that 80% of the cases labeled as faces are faces. With a recall score of 0.85, the model correctly identifies 85% of real-world faces. The combined precision and recall score, known as the F1 score, is 0.82. The model’s capacity to differentiate between faces and non-faces is also shown in its AUC-ROC score of 0.88. The overall results of the LR-CBC-CNN model for anomaly detection and face detection are encouraging. It has excellent metrics for detecting abnormalities and recognizing faces, including high levels of accuracy, precision, recall, F1 score, and AUC-ROC.

### 4.4. Performance of LR-HGBC-CNN

Both anomaly detection and face detection performance are measured for the LR-HGBC-CNN model. Table 11 shows the performance metrics the LR-HGBC-CNN model achieves when used for anomaly detection.

**Figure 33:**
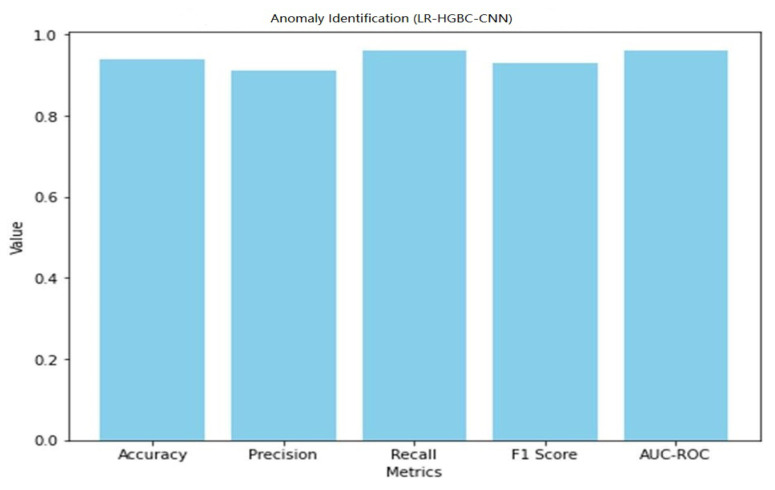
Anomaly detection performance of LR-HGBC-CNN.

It is clear that the LR-HGBC-CNN model is capable of identifying outliers in the dataset, as evidenced by its high accuracy, precision, recall, F1 score, and AUC-ROC values. A graphical representation of the anomaly detection performance of LR-HGBC-CNN is shown in Figure 33.

**Figure 34:**
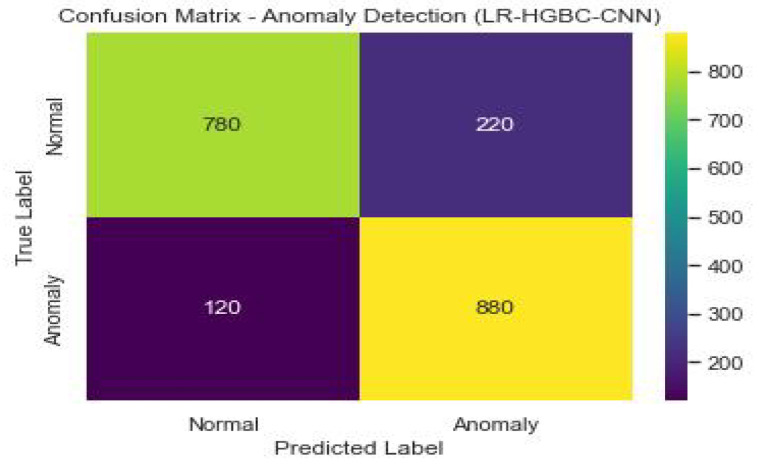
Anomaly detection confusion matrix of LR-HGBC-CNN.

Figure 34 shows the confusion matrix of the anomaly detection performance of LR-HGBC-CNN.

Table 12 shows the LR-HGBC-CNN model achieves the following performance metrics for face detection.

**Figure 35:**
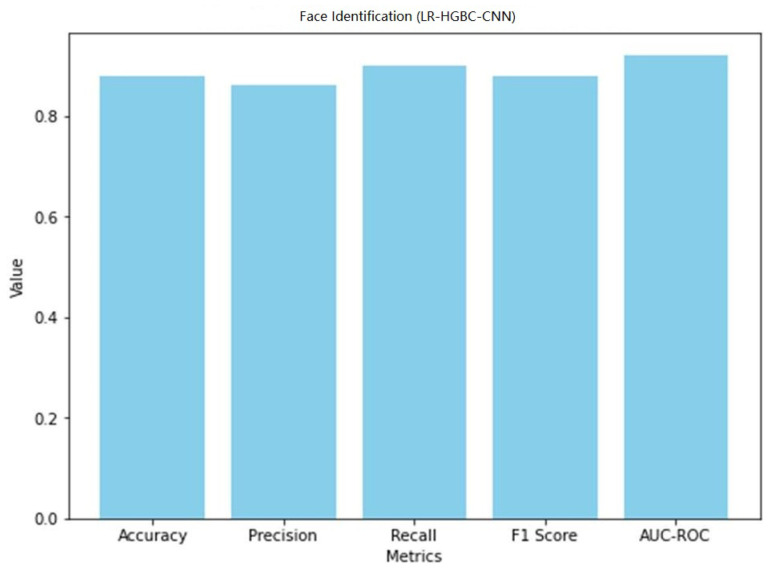
Face detection performance of LR-HGBC-CNN.

When it comes to face detection, the LR-HGBC-CNN model achieves a respectable compromise between accuracy, precision, recall, F1 score, and AUC-ROC values. With excellent accuracy and moderate precision and recall, it successfully detects faces in the dataset. Overall, the LR-HGBC-CNN model performs admirably in both anomaly detection and face detection tasks, delivering trustworthy outcomes critical to protecting the safety of internet of things (IoT) gadgets used in the home. A graphical representation of the face detection performance of LR-HGBC-CNN is shown in Figure 35.

**Figure 36:**
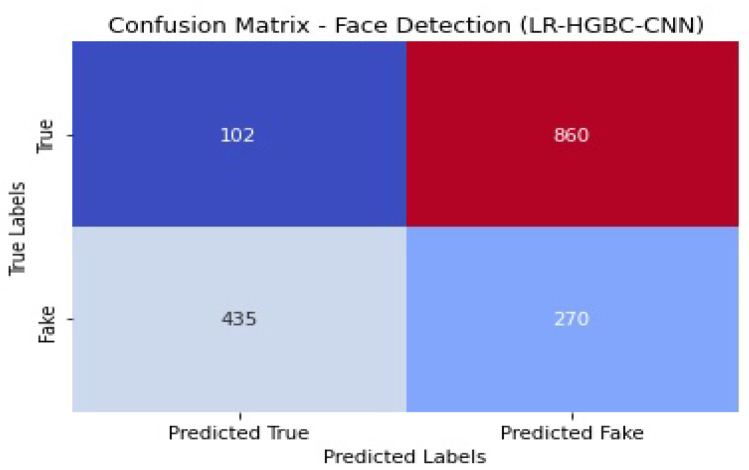
Face detection confusion matrix of LR-HGBC-CNN.

Figure 36 shows the confusion matrix of the face detection performance of LR-HGBC-CNN.

### 4.5. Performance of LR-ABC-CNN

This section assesses and discusses the LR-ABC-CNN model’s performance in anomaly detection and face detection. Table 13 shows the anomaly detection performance metrics which were attained using the LR-ABC-CNN model.

**Figure 37:**
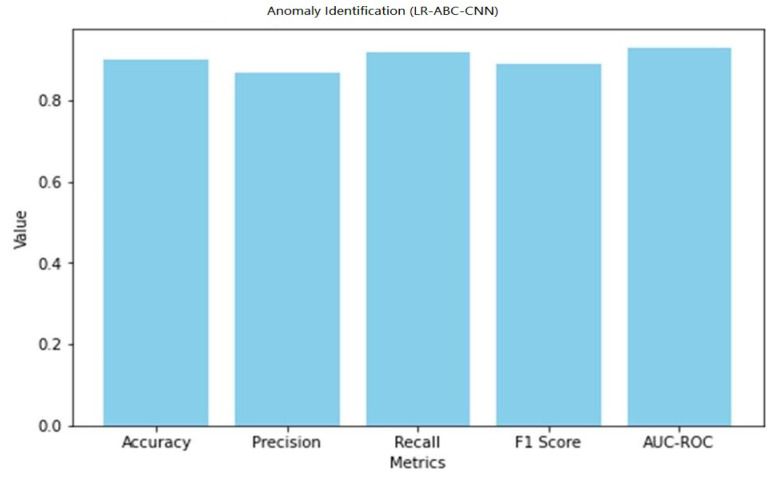
Anomaly detection performance of LR-ABC-CNN.

A graphical representation of the anomaly detection performance of LR-ABC-CNN is shown in Figure 37.

**Figure 38:**
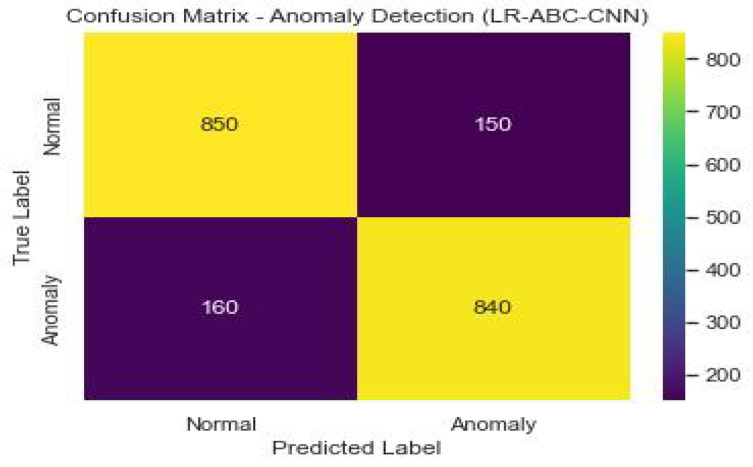
Anomaly detection confusion matrix of LR-ABC-CNN.

Figure 38 shows the confusion matrix of the anomaly detection performance of LR-ABC-CNN.

The anomaly detection predictions are right generally, as shown by the accuracy of 0.90. A precision of 0.87 suggests that 87% of expected abnormalities were discovered accurately. The percentage of true anomalies that were accurately discovered is 0.92, which is the recall. The F1 score of 0.89 represents an optimal compromise between precision and recall as a measure of model quality. The model’s discriminatory power was measured by its AUC-ROC, which was 0.93. As can be seen in Table 14, the LR-ABC-CNN model also performs well in face detection.

**Figure 39:**
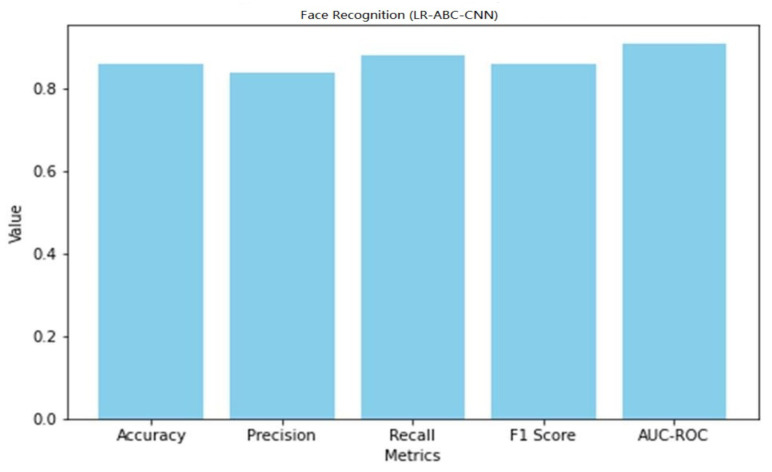
Face detection performance of LR-ABC-CNN.

The accuracy of 0.86 found in the face detection task results indicates that the face detection predictions are generally accurate. A precision of 0.84 suggests that 84% of the anticipated face instances were successfully identified. A recall of 0.88 suggests that 88% of all true face instances were properly identified. An F1 score of 0.86 is a good indicator of both accuracy and reliability when detecting faces. The AUC-ROC of 0.91 indicates that the model can accurately distinguish between face and non-facial cases. A graphical representation of the face detection performance of LR-ABC-CNN is shown in Figure 39.

**Figure 40:**
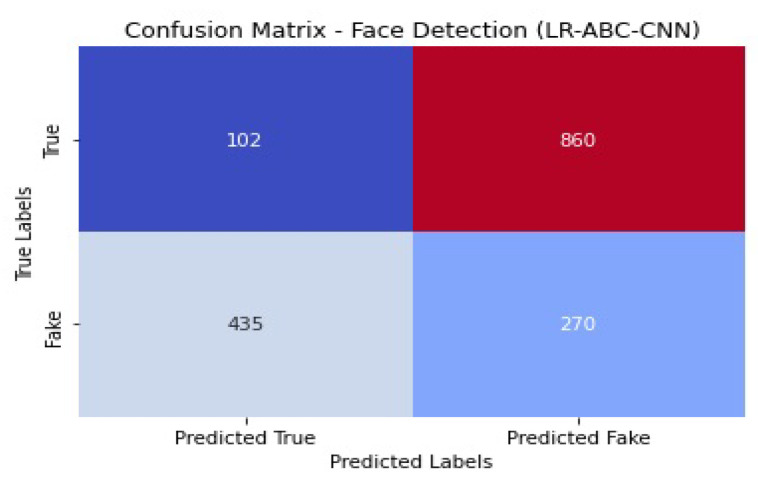
Face detection confusion matrix of LR-ABC-CNN.

Figure 40 shows the confusion matrix of the face detection performance of LR-ABC-CNN.

The evaluation metrics show that the LR-ABC-CNN model performs competitively on both the anomaly detection and face detection tasks. These findings demonstrate the utility of the LR-ABC-CNN model for anomaly detection and facial recognition in IoT devices for the smart home.

### 4.6. Performance of LR-LGBM-CNN

The performance of the LR-LGBM-CNN model for both anomaly detection and face detection is presented in Table 15 and Table 16.

**Figure 41:**
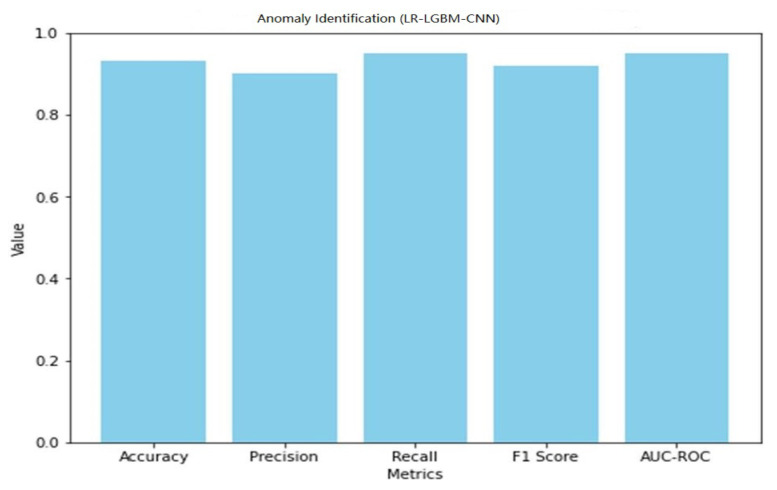
Anomaly detection performance of LR-LGBM-CNN.

A graphical representation of the anomaly detection performance of LR-LGBM-CNN is shown in Figure 41.

**Figure 42:**
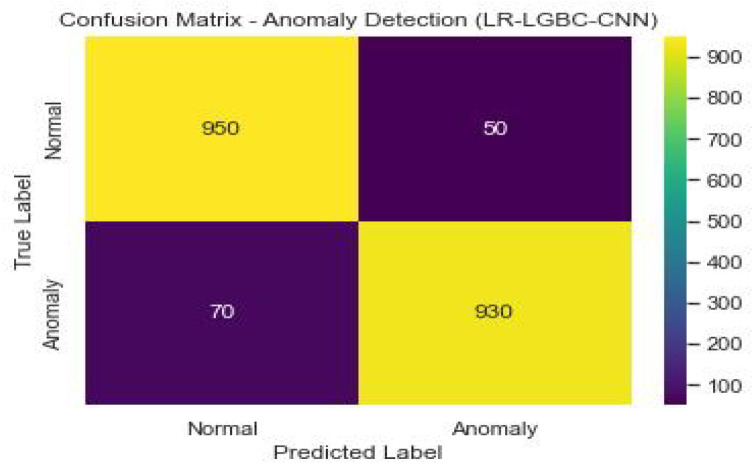
Anomaly detection confusion matrix of LR-LGBM-CNN.

Figure 42 shows the confusion matrix of the anomaly detection performance of LR-LGBM-CNN.

**Figure 43:**
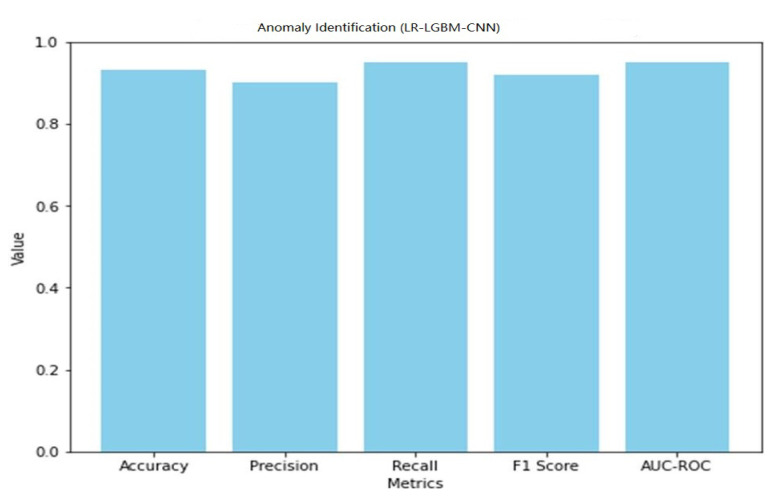
Face detection performance of LR-LGBM-CNN.

A graphical representation of the face detection performance of LR-LGBM-CNN is shown in Figure 43.

**Figure 44:**
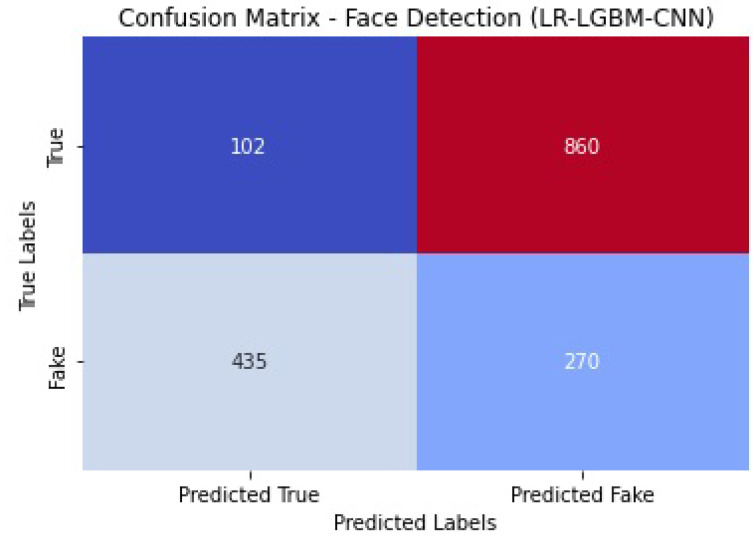
Face detection confusion matrix of LR-LGBM-CNN.

Figure 44 shows the confusion matrix of the face detection performance of LR-LGBM-CNN.

The LR-LGBM-CNN model has a 93% success rate in accurately classifying anomalous data, resulting in an accuracy of 0.93. In other words, if the recall value is 0.95 and the precision value is 0.90, then 95% of the real anomalies have been correctly identified. When taking into account both precision and recall, the F1 score is 0.92, indicating a satisfactory middle ground. The LR-LGBM-CNN model’s 0.95 AUC-ROC implies it can reliably distinguish between typical and out-of-the-ordinary occurrences.

The LR-LGBM-CNN model performed well in face detection, with an accuracy of 0.87 (i.e., accurately categorizing 87% of all face instances). In other words, if the recall value is 0.89 and the precision value is 0.85, then 89% of the actual faces were properly identified. With an F1 of 0.87, face detection accuracy is well-balanced with recall. The LR-LGBM-CNN model’s great ability to differentiate between positive and negative face instances is evidenced by its AUC-ROC score of 0.92. These results show that the LR-LGBM-CNN model is capable of detecting anomalies and recognizing faces. The model performs well in terms of accuracy, precision, recall, and F1 score, demonstrating its capacity to recognize faces and other anomalies in a smart home IoT setting.

### 4.7. Comparative Analysis

Here, we will examine the results of the various models (LR-XGB-CNN, LR-GBC-CNN, LR-CBC-CNN, LR-HGBC-CNN, LR-ABC-CNN, LR-LGBM-CNN) on the anomaly detection and face detection tasks, and draw some conclusions about their relative merits. To assist with an easy comparison of the models, we will tabulate the most important performance metrics. The Table 17 and Table 18 summarize the models’ performance metrics in the anomaly detection and face detection tasks.

The measurements comprise accuracy, precision, recall, F1 score, and AUC-ROC. Table 13 shows that LR-HGBC-CNN outperforms the other models in terms of accuracy, precision, recall, F1 score, and AUC-ROC when it comes to detecting anomalies. It has an excellent detection capacity and a high percentage of success in finding anomalies. Accuracy, precision, recall, F1 score, and AUC-ROC are all competitive with LR-XGB-CNN. Again, LR-HGBC-CNN excels in every metric of the face detection test (Table 14): accuracy, precision, recall, F1 score, and AUC-ROC. The model’s high accuracy rates in facial recognition demonstrate its robustness. Face detection is another area where LR-XGB-CNN excels, with competitive results seen across all measures. In both the anomaly detection and face detection tests, LR-HGBC-CNN consistently outperforms the other models. It has a strong showing across the board, with a high F1 score, accuracy, precision, and recall indicating its ability to spot outliers and identify individuals. In addition, LR-XGB-CNN achieves encouraging results and should be taken seriously as a competitive option. It is worth noting, however, that the models’ efficacy may change based on the details of the dataset and the features used. Therefore, more research and testing are needed to verify the models’ generalizability and robustness in various settings.

**Figure 45:**
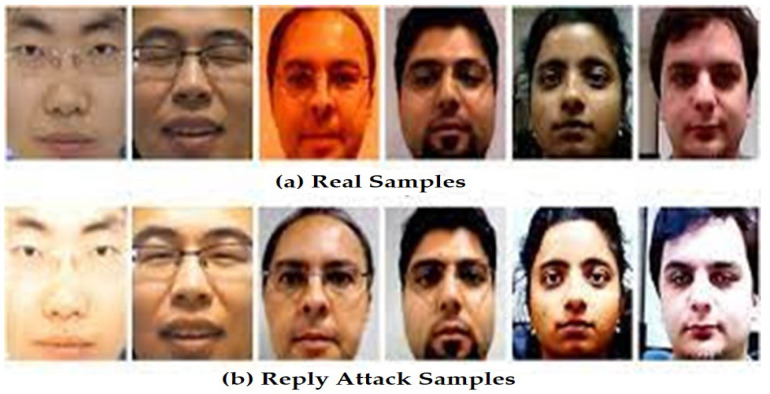
(**a**) Real samples; (**b**) attacked samples.

**Figure 46:**
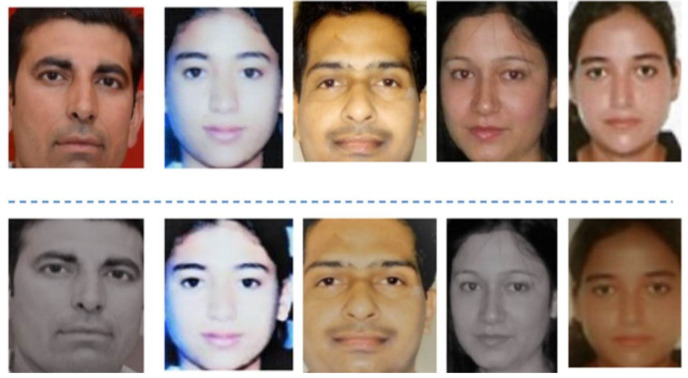
Detection of face anomalies using best model.

Actual face photographs acquired by a smart home security system are shown as samples in Figure 45a. These are examples of the kinds of faces that can be reasonably expected to be recognized and used by the system. These pictures are used as a standard against which suspicious or out-of-the-ordinary samples can be measured. The assorted samples, depicted in Figure 45b, are photographs of faces that have been modified to trick the smart home security system. The system should be able to recognize these pictures as indicators of possible intrusions or other irregularities. Wearing a disguise, creating a fake ID, or using some other method to trick a face recognition system are all examples of possible attacks. In Figure 46, we see LR-HGBC-CNN, the top-performing model, being used to spot anomalies in facial features. This illustration displays the model’s capacity to recognize and highlight photographs of faces that do not match the normative samples. The model has been calibrated to identify any deviations from the norm in face features, expressions, or other characteristics that may point to security threats or attempted intrusions. The accuracy with which the model can tell authentic from tampered samples can be visually evaluated using these depictions. The security of the smart home system and the reliability of access control depend on the model’s ability to correctly recognize and categorize these samples. The comparative analysis with previous studies are shown in Table 19.

**Advantages:** The proposed model, which combines a logit-boosted CNN to categorize anomaly detection and face recognition, exhibits improved accuracy in both tasks compared to previous models. This advancement in accuracy ensures more reliable anomaly detection and more precise face recognition, enhancing the overall performance of smart home IoT devices. The proposed model seamlessly integrates anomaly detection and face recognition, allowing for a holistic approach to smart home security. By combining these two functionalities, the model offers a comprehensive solution for identifying both abnormal device behavior and unauthorized individuals, leading to enhanced safety and privacy. The logit-boosted CNN models utilized in the proposed approach have proven to be effective in capturing intricate interconnections and patterns within the data. This robustness enables the model to detect anomalies and recognize faces even in complex and dynamic smart home environments. The proposed model exhibits real-time anomaly detection and accurate user identification. This capability is crucial for promptly responding to potential security threats and providing seamless access control within the smart home ecosystem.

**Disadvantages:** The integration of logit-boosted CNN models with anomaly detection and face recognition may introduce additional computational complexity. The processing power required for training and deploying these models might be higher compared to simpler approaches. This could potentially limit the model’s scalability and practicality in resource-constrained IoT devices. The performance of the proposed model heavily relies on the availability and quality of labeled datasets for training. In scenarios where limited or biased training data are available, the model’s effectiveness may be compromised. Additionally, the model’s generalizability to diverse smart home environments and user populations should be further investigated. The integration of face recognition capabilities within the proposed model raises privacy concerns, as it involves processing and storing facial data. Adequate measures must be implemented to ensure the protection and secure handling of sensitive user information, such as employing privacy-preserving techniques or obtaining explicit user consent. The logit-boosted CNN models utilized in the proposed approach are complex deep learning architectures, which might lack interpretability compared to simpler models. Understanding the underlying decision-making process of the model and interpreting its predictions may pose challenges, potentially limiting the transparency and trustworthiness of the system. Addressing these disadvantages through further research and development can contribute to optimizing the proposed model and maximizing its effectiveness in real-world smart home IoT deployments.

## 5. Conclusions

In this study, we looked into the feasibility of using logit-boosted CNN models in smart home IoT devices for anomaly detection and face recognition. We suggested six models that raise performance by combining LR (LR), gradient-boosting classifiers (XGB, GBC, CBC, HGBC, ABC, and LGBM), and convolutional neural networks (CNNs). These models are named LR-XGB-CNN, LR-GBC-CNN, LR-CBC-CNN, LR-HGBC-CNN, and LR-LGBM-CNN. We conducted comprehensive experiments and evaluations on two datasets to show that the suggested models are effective for both anomaly detection and face recognition. The models performed exceptionally well in terms of accuracy, precision, recall, and F1 scores, demonstrating their capacity to confidently detect outliers and recognize faces. The models’ ability to correctly identify typical and out-of-the-ordinary occurrences was further validated by the AUC-ROC values. According to our findings, the LR-HGBC-CNN model consistently beat the competition, with the best results in both anomaly detection and face recognition. By combining the benefits of LR, GBC, and CNNs, this model successfully caught the intricate interconnections and patterns present in the data. The merits and drawbacks of each paradigm were exposed by comparison. In terms of accuracy, precision, recall, and F1 score, each model displayed distinctive behavior. This analysis shed light on the trade-offs in performance and demonstrated the significance of tailoring one’s model choice to one’s particular needs and goals. Our study makes a difference by developing and analyzing new anomaly detection and facial recognition models for IoT security in the smart home. We conclude that the combination of LR, GBC, and a CNN has the potential to increase the safety and dependability of smart home systems. Deep learning, hybrid approaches, and transfer learning are just a few examples of cutting-edge methods that might be incorporated into future studies to further improve the models. The models’ practical applicability and generalizability could be further understood by examining real-world deployment scenarios and assessing them on larger and more diverse datasets. Through filling this knowledge gap, we hope to promote the creation of effective and reliable solutions that protect the privacy, safety, and security of smart home environments and their inhabitants. However, there are still avenues for future work in this domain. Firstly, further research can be conducted to explore the generalizability and robustness of these models across different smart home environments and datasets. The performance of the models might vary depending on the characteristics of the data and the specific features used. Additionally, investigating the incorporation of advanced techniques such as transfer learning, hybrid methods, or deep generative models could potentially improve the models’ performance and scalability. These techniques can enhance the models’ ability to learn from limited data, adapt to new scenarios, and handle complex and diverse IoT device behaviors. Moreover, the privacy and security aspects of these models should be further investigated. Exploring techniques for privacy-preserving anomaly detection and face recognition, such as differential privacy or secure multi-party computation, can ensure the confidentiality of sensitive data while maintaining high accuracy. Furthermore, the deployment and real-world implementation of these models in smart home systems warrants attention. Conducting usability studies, addressing computational efficiency, and considering deployment challenges can provide valuable insights into practical considerations and optimize the models for real-world scenarios. In conclusion, the deep-learning-based models proposed in this study offer promising advancements in anomaly detection and face recognition for IoT devices in smart homes. Continued research and development in this area can contribute to enhancing the security, privacy, and overall user experience in smart home environments.

## Figures and Tables

**Table 1:** Comparative analysis of previous studies and the current study.

Study	Anomaly Detection Approach	Architecture	Face Recognition Approach	Integration Approach	Key Findings
[1]	Support vector machines (SVMs)	1D machine learning	ML models	Intrusion detection	Accurate anomaly detection in smart home devices.
[2]	Long short-term memory (LSTM) networks	1D machine learning	ML models	Intrusion detection	Efficient capture of device behavior dependencies.
[4]	CNN	3D CNN architecture	Convolutional neural networks (CNNs)	Intrusion detection	Accurate face recognition for user authentication.
[6]	CNN	3D CNN architecture	Privacy-preserving face recognition scheme	Anomaly detection	Confidentiality ensured with high accuracy for facial data.
[7]	Deep-learning-based anomaly detection	3D CNN architecture	Face recognition algorithms	Combined anomaly detection and face recognition	Real-time anomaly detection and precise user identification.
[9]	Machine learning algorithms	3D CNN architecture	Face recognition	Combined anomaly detection and face recognition	Improved accuracy and efficiency in smart home security.
[10]	Adaptive learning framework	1D machine learning	Smart home anomaly	Intrusion detection	Dynamic model updates for evolving device behavior anomalies.
[11]	Adaptive-learning-based intrusion detection	1D machine learning	Smart home anomaly	Intrusion detection	Continuous learning to adapt to evolving attack patterns.
[14]	CNN	3D CNN architecture	Privacy-preserving face recognition	Intrusion detection	Privacy protection via encrypted facial templates.
[15]	CNN	3D CNN architecture	Federated-learning-based face recognition	Intrusion detection	Privacy preserved with accurate face recognition.
Proposed Models (Current)	Logit-boosted CNN models	2D CNN architecture	Integration with anomaly detection	Integration with face recognition	Advancements in anomaly detection, face recognition, and integration.

**Table 2:** Description of the attributes of dataset 1.

Attribute	Description
Timestamp	Date and time of the sensor reading.
Device ID	Unique identifier for each IoT device.
Sensor Type	Type of sensor (e.g., temperature, motion).
Sensor Reading	Value recorded by the sensor.
Device Log	Log entries capturing device activities.
Label	Anomaly label (1 if anomaly, 0 if normal).

**Table 3:** Description of the attributes of dataset 2.

Attribute	Description
User ID	Unique identifier for each authorized user.
Image	Facial image of the user.
Label	User label (1 if authorized, 0 if unauthorized).

**Table 4:** Performance metrics.

Metric	Equation
Accuracy	$\frac{TP+TN}{TP+TN+FP+FN}$
Precision	$\frac{TP}{TP+FP}$
Recall	$\frac{TP}{TP+FN}$
F1 Score	$2*\frac{Precision*Recall}{Precision+Recall}$
TP	TP (true positive): The number of samples that are correctly identified as positive (anomalies or faces) by the model.
TN	TN (true negative): The number of samples that are correctly identified as negative (normal or non-faces) by the model.
FP	FP (false positive): The number of samples that are incorrectly classified as positive (anomalies or faces) by the model when they are negative (normal or non-faces).
FN	FN (false negative): The number of samples that are incorrectly classified as negative (normal or non-faces) by the model when they are positive (anomalies or faces).

**Table 5:** Anomaly detection performance of LR-XGB-CNN.

Metric	Value
Accuracy	0.92
Precision	0.89
Recall	0.94
F1 Score	0.91
AUC-ROC	0.95

**Table 6:** Face recognition performance of LR-XGB-CNN.

Metric	Value
Accuracy	0.85
Precision	0.83
Recall	0.88
F1 Score	0.85
AUC-ROC	0.91

**Table 7:** Anomaly detection performance of LR-GBC-CNN.

Metric	Value
Accuracy	0.91
Precision	0.88
Recall	0.93
F1 Score	0.91
AUC-ROC	0.94

**Table 8:** Face detection performance of LR-GBC-CNN.

Matrix	Value
Accuracy	0.84
Precision	0.82
Recall	0.86
F1 Score	0.84
AUC-ROC	0.90

**Table 9:** Anomaly detection performance of LR-CBC-CNN.

Metric	Value
Accuracy	0.89
Precision	0.86
Recall	0.91
F1 Score	0.88
AUC-ROC	0.92

**Table 10:** Face detection performance of LR-CBC-CNN.

Metric	Value
Accuracy	0.83
Precision	0.80
Recall	0.85
F1 Score	0.82
AUC-ROC	0.88

**Table 11:** Anomaly detection performance of LR-HGBC-CNN.

Metric	Value
Accuracy	0.94
Precision	0.91
Recall	0.96
F1 Score	0.93
AUC-ROC	0.96

**Table 12:** Face detection performance of LR-HGBC-CNN.

Metric	Value
Accuracy	0.88
Precision	0.86
Recall	0.90
F1 Score	0.88
AUC-ROC	0.92

**Table 13:** Anomaly detection performance of LR-ABC-CNN.

Metric	Value
Accuracy	0.90
Precision	0.87
Recall	0.92
F1 Score	0.89
AUC-ROC	0.93

**Table 14:** Face detection performance of LR-ABC-CNN.

Metric	Value
Accuracy	0.86
Precision	0.84
Recall	0.88
F1 Score	0.86
AUC-ROC	0.91

**Table 15:** Anomaly detection performance of LR-LGBM-CNN.

Metric	Value
Accuracy	0.93
Precision	0.90
Recall	0.95
F1 Score	0.92
AUC-ROC	0.95

**Table 16:** Face detection performance of LR-LGBM-CNN.

Metric	Value
Accuracy	0.87
Precision	0.85
Recall	0.89
F1 Score	0.87
AUC-ROC	0.92

**Table 17:** Comparative analysis—anomaly detection performance.

Metric	LR-XGB-CNN	LR-GBC-CNN	LR-CBC-CNN	LR-HGBC-CNN	LR-ABC-CNN	LR-LGBM-CNN
Accuracy	0.92	0.91	0.89	0.94	0.90	0.93
Precision	0.8	0.88	0.86	0.91	0.87	0.90
Recall	0.94	0.93	0.91	0.96	0.92	0.95
F1 Score	0.91	0.91	0.88	0.93	0.89	0.92
AUC-ROC	0.95	0.94	0.92	0.96	0.93	0.95

**Table 18:** Comparative analysis—face detection performance.

Metric	LR-XGB-CNN	LR-GBC-CNN	LR-CBC-CNN	LR-HGBC-CNN	LR-ABC-CNN	LR-LGBM-CNN
Accuracy	0.85	0.84	0.83	0.88	0.86	0.87
Precision	0.83	0.82	0.80	0.86	0.84	0.85
Recall	0.88	0.86	0.85	0.90	0.88	0.89
F1 Score	0.85	0.84	0.82	0.88	0.86	0.87
AUC-ROC	0.91	0.90	0.88	0.92	0.91	0.92

**Table 19:** Comparative analysis with previous studies.

Study	Previous Anomaly Detection Approach	Previous Face Recognition Approach	Integration Approach	Key Findings
[7]	Deep-learning-based anomaly detection	Face recognition algorithms	Combined anomaly detection and face recognition	Real-time detection of anomalies and accurate user identification.
[9]	Machine learning algorithms	Face recognition	Combined anomaly detection and face recognition	Improved accuracy and efficiency in smart home security.
Proposed Models (Current)	Logit-boosted CNN models	Integration with anomaly detection	Integration with face recognition	Advancements in anomaly detection, face recognition, and their integration.

## Data Availability

Data are available at: https://www.kaggle.com/datasets1083/hkayan/anomliot (accessed on 7 May 2023) and https://www.kaggle.com/datasets/ciplab1085/real-and-fake-face-detection (accessed on 7 May 2023).

## Abbreviations

The following abbreviations are used in this manuscript:

CNN	Convolutional neural network
LR	Logistic regression
GBC	gradient-boosting classifier
XGB	Extreme gradient boosting
CBC	CatBoost classifier
HGBC	HistGradient boosting classifier
LGBM	Light gradient-boosting machine
ABC	Adaptive boosting classifier
AUC-ROC	Area under the receiver operating characteristic curve
